# Transcranial photobiomodulation for brain diseases: review of animal and human studies including mechanisms and emerging trends

**DOI:** 10.1117/1.NPh.11.1.010601

**Published:** 2024-02-05

**Authors:** Hao Lin, Dongyu Li, Jingtan Zhu, Shaojun Liu, Jingting Li, Tingting Yu, Valery V. Tuchin, Oxana Semyachkina-Glushkovskaya, Dan Zhu

**Affiliations:** a Huazhong University of Science and Technology, Britton Chance Center for Biomedical Photonics, Wuhan National Laboratory for Optoelectronics – Advanced Biomedical Imaging Facility, Wuhan, China; b Huazhong University of Science and Technology, School of Optical Electronic Information, Wuhan, China; c Huazhong University of Science and Technology, School of Engineering Sciences, Wuhan, China; d Saratov State University, Science Medical Center, Saratov, Russia; e Research Center of Biotechnology of the Russian Academy of Sciences, Bach Institute of Biochemistry, Moscow, Russia; f Tomsk State University, Laboratory of Laser Molecular Imaging and Machine Learning, Tomsk, Russia; g Humboldt University, Department of Physics, Berlin, Germany

**Keywords:** transcranial photobiomodulation, low-level laser (light) therapy, mechanisms, brain disease

## Abstract

The brain diseases account for 30% of all known diseases. Pharmacological treatment is hampered by the blood–brain barrier, limiting drug delivery to the central nervous system (CNS). Transcranial photobiomodulation (tPBM) is a promising technology for treating brain diseases, due to its effectiveness, non-invasiveness, and affordability. tPBM has been widely used in pre-clinical experiments and clinical trials for treating brain diseases, such as stroke and Alzheimer’s disease. This review provides a comprehensive overview of tPBM. We summarize emerging trends and new discoveries in tPBM based on over one hundred references published in the past 20 years. We discuss the advantages and disadvantages of tPBM and highlight successful experimental and clinical protocols for treating various brain diseases. A better understanding of tPBM mechanisms, the development of guidelines for clinical practice, and the study of dose-dependent and personal effects hold great promise for progress in treating brain diseases.

## 1 Introduction

The brain is a vital component of the central nervous system (CNS) and is in charge of an individual’s life activities, such as thinking, emotion, and memory. Brain regions are anatomically interconnected, exhibiting both specialization and collaboration in their functions, making it one of the most intricate and advanced systems found in nature. However, the brain is extremely vulnerable to various disorders, including cerebrovascular diseases, neurodegenerative diseases, brain inflammation, etc.,^1^^–^^4^ which have posed a significant burden on individuals, families, and society as a whole.

Drug therapy is the most common therapeutic approach for brain diseases in clinical practice. However, drug therapy may produce adverse side effects, and the highly selective permeability of the blood–brain barrier (BBB) significantly limits brain drug delivery, resulting in a disappointing therapeutic effect.^5^ Some novel drug delivery strategies, such as viral vectors and non-viral nanoparticles, have demonstrated prodigious potential for delivering therapeutic to the brain. However, the safety of injecting a virus and the penetrability of the nanoparticles and their encapsulated payloads require further study.^6^ Recently, erythropoietin (EPO), stem cell therapy, transcranial magnetic stimulation (TMS), and transcranial direct current stimulation (tDCS) have been proposed as potential therapeutics for brain diseases.^7^^–^^10^ However, EPO naturally increases the hematocrit, which could heighten the risk of cardiovascular reperfusion.^11^ For stem-based therapies, the ethical issues and safety concerns need further discussion.^12^ TMS and tDCS lack clear mechanisms underlying their therapeutic effects, and the absence of widely accepted guidelines and standards for treating brain diseases impedes their clinical applications.^13^^,^^14^ Therefore, development of a safe, effective, and widely used therapeutic approach for brain diseases is urgent.

**Fig. 1:**
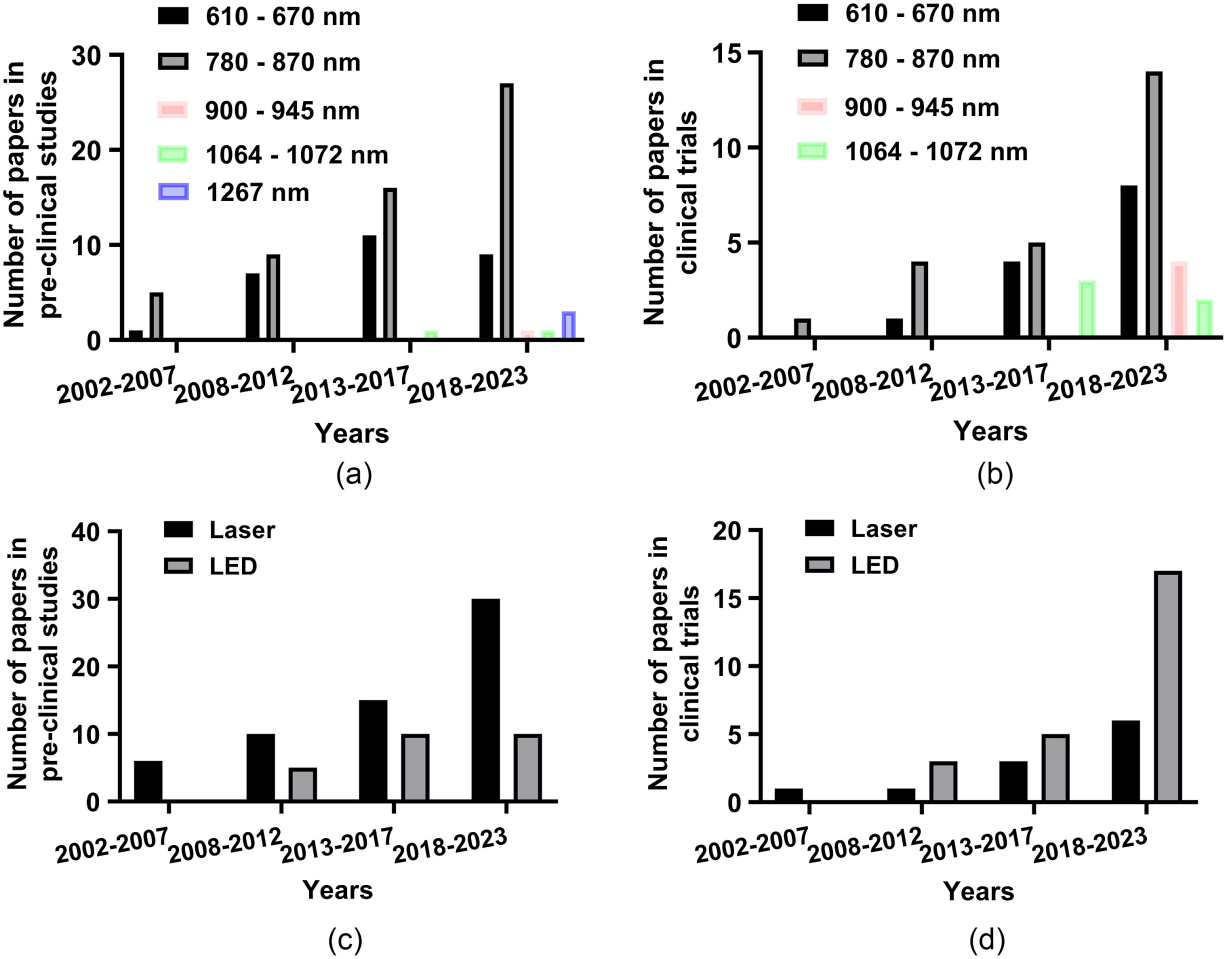
Number of published articles using different tPBM wavelengths and light sources for brain disease treatment in pre-clinical studies and clinical trials from 2002 to 2023. (a) The number of published articles using different tPBM wavelengths in pre-clinical studies. (b) The number of published articles using different tPBM wavelengths in clinical trials. (c) The number of published articles using different tPBM sources in pre-clinical studies. (d) The number of published articles using different tPBM sources in clinical trials. Notes: the data are summarized from Refs. 20–23 and 51–70, (ischemic stroke), 71–73 (HI), 74 (intracerebral hemorrhage), 25–28, 75–83 (AD), 29–34, 84–96 (PD), 97–99 (multiple sclerosis), 35–40, 100–116 (TBI), 117 (possible chronic traumatic encephalopathy), 41–44, 118–126 (depression), 45–50, 127–136 (aging), and 137–141 (epilepsy).

**Fig. 2:**
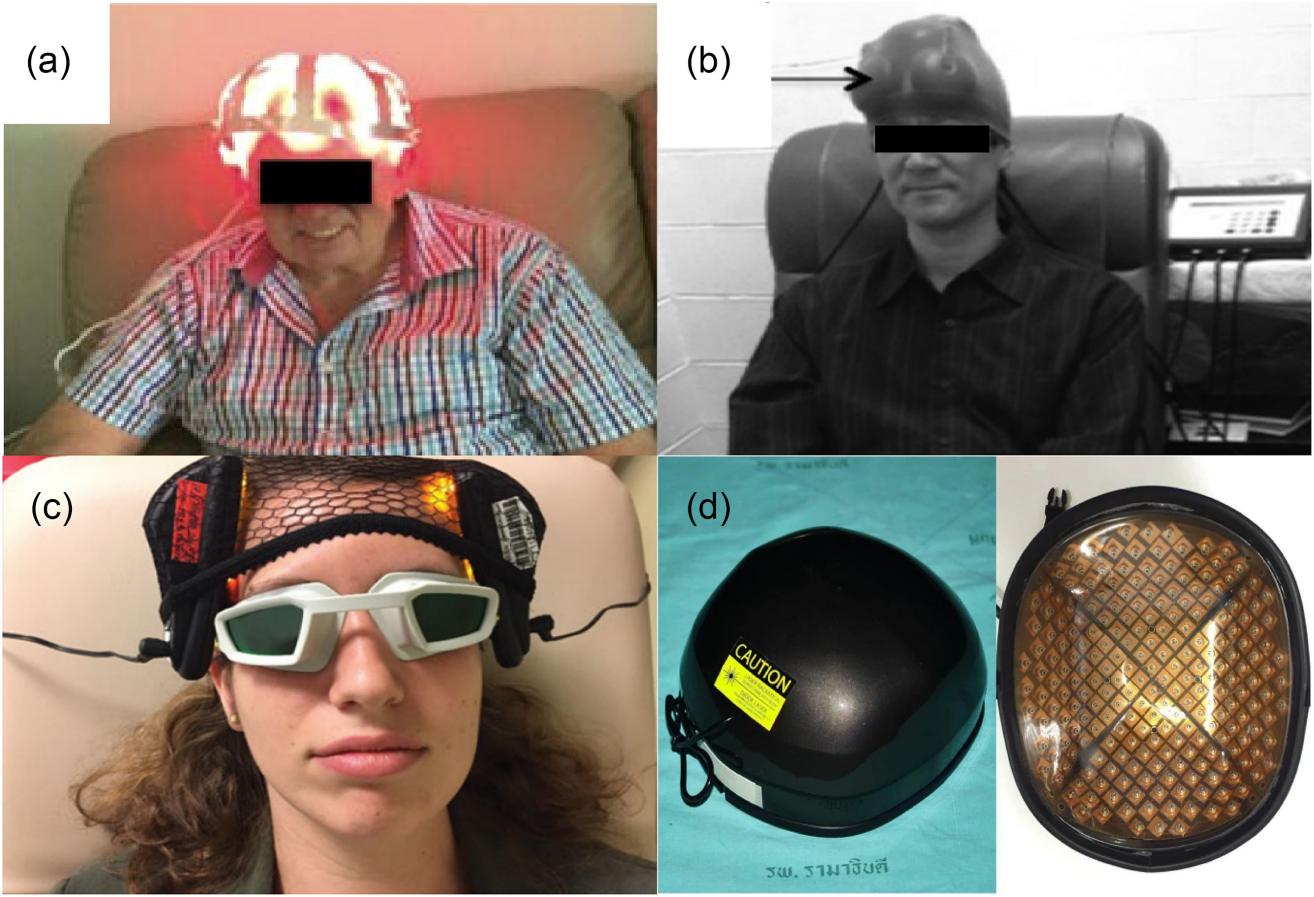
Examples of tPBM helmet devices for clinical treatment of brain diseases: (a) PD,^31^ (b) TBI,^36^ (c) depression,^43^ and (d) androgenetic alopecia.^160^

Transcranial photobiomodulation (tPBM), a non-invasive and non-thermal brain stimulation therapy, was proposed over 50 years ago. Numerous related devices are also available in the market. tPBM was known as low-level laser (light) therapy at the early stage, but more and more researchers prefer tPBM in recent years because it is more indicative of its scientific principle.^15^ tPBM refers to applying low irradiance (0.01 to $10\;W/{\mathrm{cm}}^{2}$) red to near-infrared (NIR) (600 to 1300 nm) light^16^^,^^17^ through the skull directly to brain tissue to achieve neuroprotection, behavioral improvement, and so on. One of the most recognized molecular mechanisms of tPBM is that cytochrome c oxidase (CCO) may dissociate inhibitory nitric oxide (NO) after absorbing photons, thereby enhancing mitochondrial activity and promoting ATP biosynthesis.^18^ Because brain disorders are closely related to mitochondrial activity, tPBM may have beneficial effects on various brain diseases.^19^ During the past two decades, a tremendous number of pre-clinical experiments with animals and clinical trials with humans have demonstrated beneficial effects. Clinical trials with humans include, in particular, the treatment of ischemic stroke,^20^^–^^24^ Alzheimer’s disease (AD),^25^^–^^28^ Parkinson’s disease (PD),^29^^–^^34^ traumatic brain injury (TBI),^35^^–^^40^ depression,^41^^–^^44^ aging,^45^^–^^50^ etc. In these studies, the parameters of the light used are extremely sophisticated and deserve to be deliberated. For wavelength, researchers prefer using 808 nm-light [Figs. 1(a) and 1(b)], which has a strong absorption peak of CCO with an excellent penetration depth.^142^ Furthermore, there also exist extensive studies that have applied other wavelengths (e.g., 610, 1070, 1267 nm) of light for brain disease treatment for specific biological targets including but not limited to mitochondrial stimulation.^51^^,^^74^^,^^75^^,^^143^ In particular, compared with traditional laser therapy (630 to 900 nm), the light in the NIR-II region (1000 to 1700 nm) has much less scattering and thus can penetrate deeper into the brain despite its slighter stronger absorption.^144^^–^^146^ Researchers have demonstrated that the light in the NIR-II region presents the absence of carcinogenic or mutagenic properties.^143^ The utilization of 1267 nm tPBM represents a burgeoning strategy in the therapeutic landscape of brain diseases, attributable to its recently unveiled unique capacity to stimulate the brain waste removal system (BWRS), thus contributing to neuroprotection of the CNS.^147^ It has been reported that such a wavelength can activate the generation of singlet oxygen in biological tissues,^148^^,^^149^ which can lead to vascular responses and intense elimination of toxins and unnecessary molecules from the brain.^150^^–^^152^ In addition, pulsed wave (PW) light has been found to penetrate deeper into tissues than continuous wave (CW) light with the same average power, as supported by theories and *ex vivo* experiments.^153^^–^^156^ Moreover, PW light with a specific frequency may activate specific ion channels (e.g. transient receptor potential channel) to trigger a series of beneficial biological responses.^75^ For the light source, whether a coherent monochromatic laser is superior to incoherent light emitting diodes (LEDs) is an ongoing debate, with some researchers affirming^157^^,^^158^ but some denying.^159^ Although lasers were preferred at the early stage, especially for preclinical trials, LEDs are widely designed as helmet-type devices for clinical trials due to their low price and easy assembly [Figs. 1(c) and 1(d)]. Figure 2 shows representative tPBM devices for treating various brain diseases, including PD,^31^ TBI,^36^ depression,^43^ and androgenetic alopecia.^160^

In this review, we shed light on the tPBM-mediated mechanisms of therapy for brain diseases according to the emerging trends in tPBM and new discoveries. We also review the animal experiments and clinical trials in different brain diseases, with the aim of providing guidance for future experimental design and clinical applications.

## 2 Main Mechanisms of tPBM

### 2.1 Molecular and Cellular Mechanisms of tPBM

**Fig. 3:**
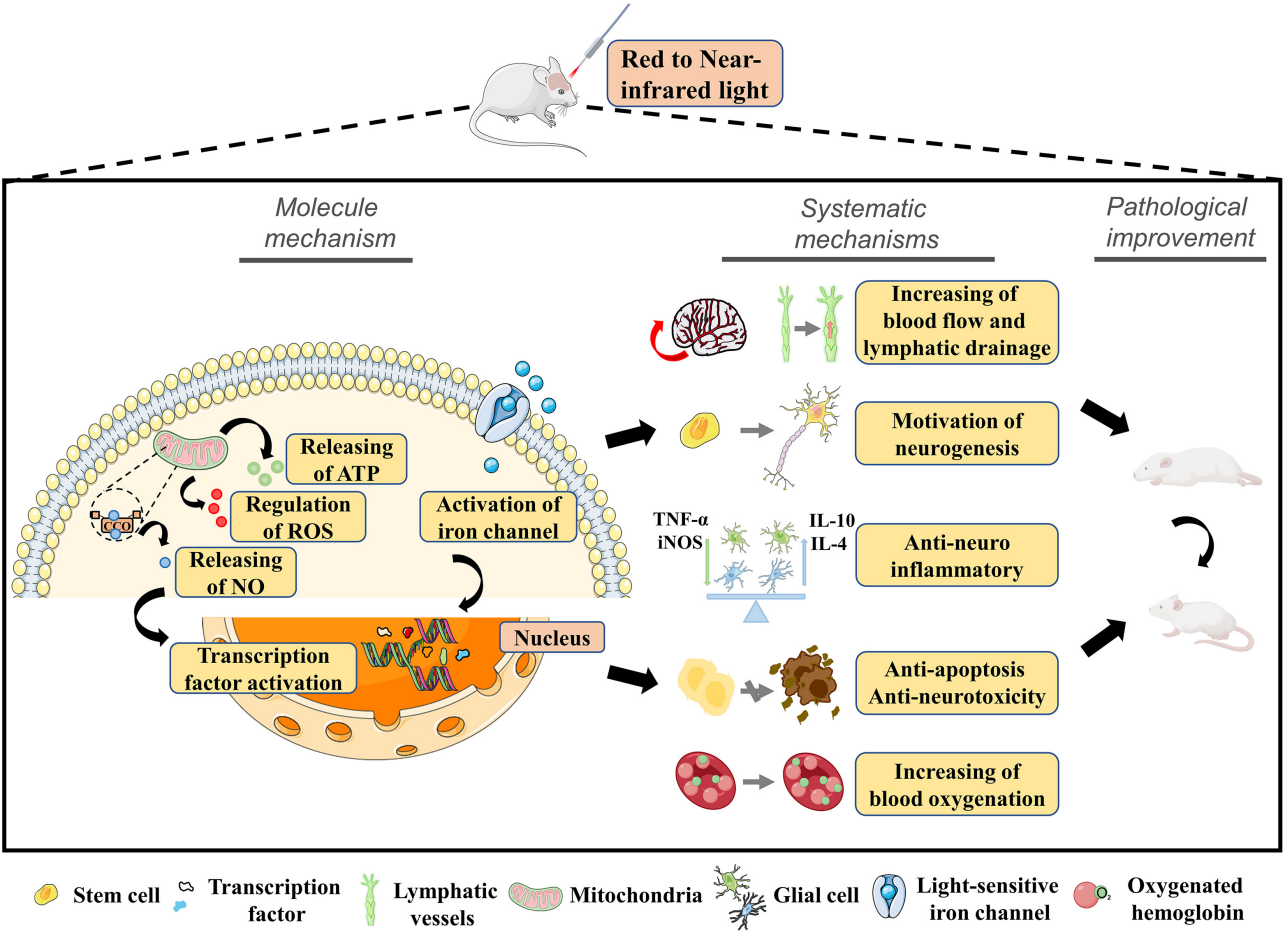
Molecular mechanisms and neurobiology effects of tPBM.

The molecular and cellular mechanisms of tPBM are very complex (Fig. 3), with the enhancement of mitochondrial activity recognized as one of the most widely studied and crucial mechanisms.^161^^,^^162^ Mitochondrial CCO is the terminal enzyme in the mitochondrial respiratory chain that contributes an increase in metabolic and energetic activity of cells via more oxygen consumption. Both *in vitro*^163^ and *in vivo*^47^^,^^164^^–^^166^ studies have revealed that red and NIR light (e.g., 660, 808, 850, and 1064 nm) can significantly increase the CCO activity and expression level. After boosting CCO, the mitochondrial membrane potential (MMP) is increased, more oxygen is consumed, more glucose is metabolized, and more ATP is produced.^167^ Furthermore, a recent study proposed that 1267 nm laser-generated singlet oxygen can work as an activator of mitochondrial respiration and ATP production in brain cells.^168^ The above evidence demonstrates that tPBM can increase cell respiration, boost brain energy metabolic capacity, enhance brain electrophysiological oscillation (alpha and beta bands) strength, and improve cerebral oxygenation,^169^^–^^172^ which would constitute an adaptation with major neuroprotective implications after brain disease.

Reactive oxygen species (ROS) is an umbrella term for an array of derivatives of molecular oxygen. It was reported that the release of low-level mitochondrial ROS is involved in regulating transcription factors and signaling mediators, potentially leading to beneficial effects.^173^ Meanwhile, downregulation of excessive ROS may also help inhibit oxidative stress and neuroinflammation, thereby protecting the neuronal mitochondria. Currently, the regulatory effects of parenchymal border macrophages (PBM) on ROS are still controversial. Some studies have shown that PBM enhanced the ROS production by activating the superoxide converting system,^174^^,^^175^ whereas other studies have suggested the opposite.^176^ A recent study found that PBM increased ROS levels in normal neurons but reduced it in oxidatively stressed neurons.^177^

The brain diseases are typically accompanied by oxidative stress damage. Therefore, reducing ROS levels may provide a potential possibility for brain disorder treatment. Salehpour et al. found that tPBM (810 nm) reduced the mitochondrial ROS levels and mitigated learning and memory impairments in aging^127^ and in ischemic stroke^128^ mouse models. Zhang’s team found that tPBM (808 nm) significantly inhibited the oxidative damage induced by the increase of superoxide anion after beta-amyloid (Aβ) intraventricular injection and significantly enhanced the total antioxidant capacity.^76^ In addition, they observed that tPBM (808 nm) increased the total antioxidant capacity in a TGF344 AD transgenic rat model and reduced the level of oxidative stress products (e.g., lipid peroxide malondialdehyde).^77^ Similar beneficial effects mediated by tPBM have also been shown in mice with sleep deprivation and depression.^118^^,^^119^

Neuroinflammation is one of the crucial pathophysiological conditions associated with various brain disorders. tPBM can decrease the expression of pro-inflammatory cytokines via inhibition of NF-κB signaling pathways.^178^ It can also directly alter the phenotype of glial cell and then regulate the release of inflammatory cytokines. Specifically, tPBM reduces the number of cortical pro-inflammatory phenotype (M1) microglia in ischemic stroke and brain trauma mouse models.^52^^,^^100^ In addition, some studies have demonstrated that tPBM promotes the polarization of cortical and hippocampal microglia from M1 to an anti-inflammatory phenotype (M2) in AD^77^ and neonatal hypoxic-ischemic (HI) rat models.^71^ Furthermore, tPBM can improve the lymph flow in the meningeal lymphatic vessels (MLVs), thereby promoting removal of pro-inflammatory cytokines from the brain.^179^

In addition to the mechanisms mentioned above, other molecular mechanisms such as gating of the channelrhodopsins and the regulation of the transcription factor hypoxia-inducible factor (HIF-1α) may also contribute to the beneficial effects of tPBM.^180^ In summary, the interaction between photons and tissues is highly complex, and the mechanism of tPBM is not yet well understood. Further exploration of the mechanism of tPBM will have important implications for clinical treatment of brain diseases.

### 2.2 Systemic Mechanisms of tPBM

**Fig. 4:**
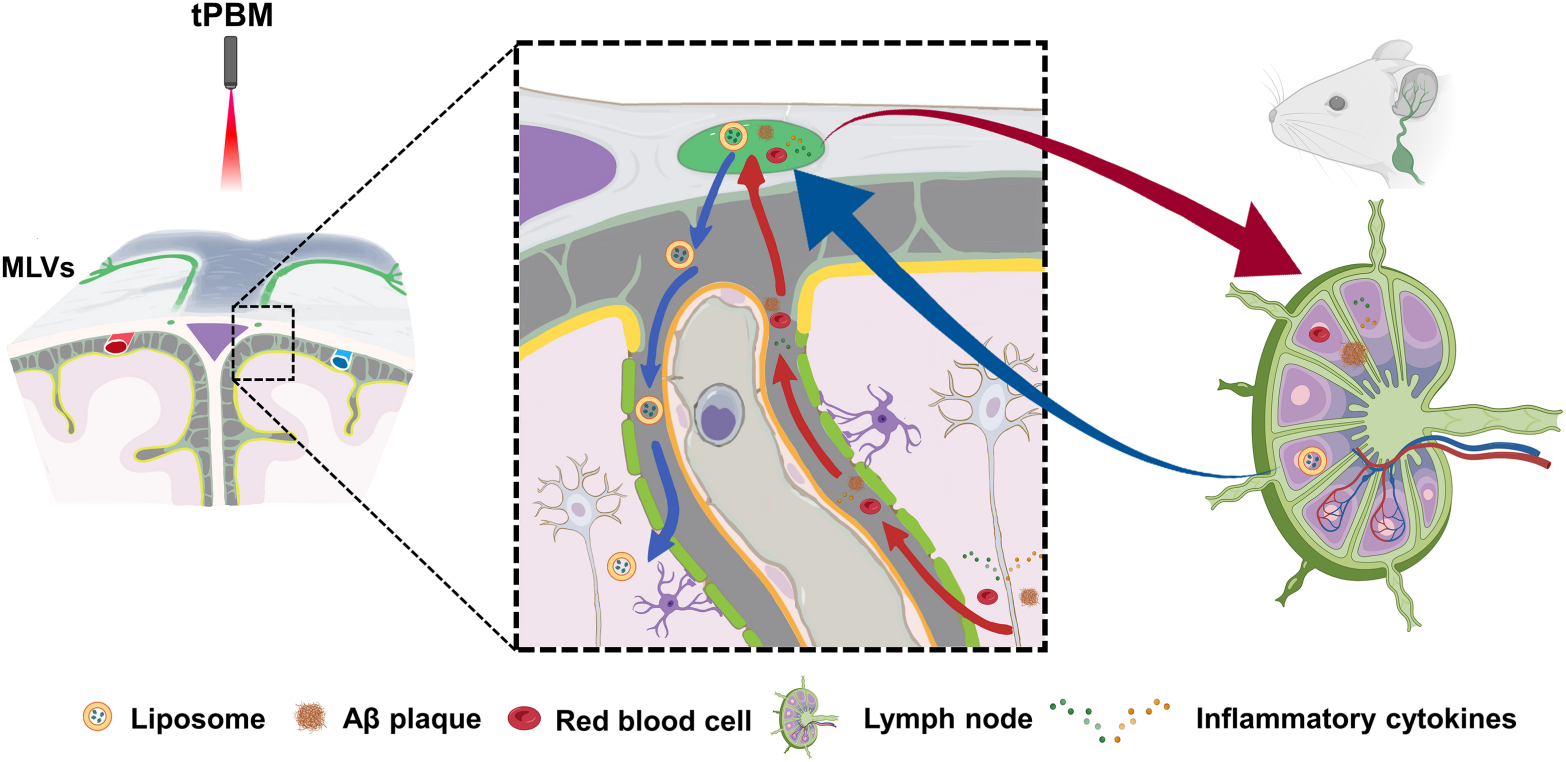
Illustration of new tPBM strategy for brain disease treatment: red and blue arrows represent MLVs-mediated toxins evacuation pathway and liposome-loaded drug delivery pathway, respectively. Some items were created with BioRender.

Recently, new effects of tPBM on the BWRS have been discovered via tPBM-induced stimulation of MLVs.^78^^,^^79^^,^^147^^,^^181^^–^^185^ *In vivo* and *in vitro* experiments have shown that PBM induces relaxation of lymphatic endothelium of both the MLVs and the mesenteric lymphatic vessels (LVs).^184^ PBM also increases the permeability of lymphatic endothelium due to a decrease in the expression of the tight junction (TJ) proteins.^184^ These effects of PBM might be related to a PBM-related stimulation of the NO synthesis in the lymphatic endothelium.^17^^,^^74^^,^^186^ The NO causes the relaxation of the blood and LVs through soluble guanylate cyclase and protein kinase G, leading to reducing of the ${\mathrm{Ca}}^{2+}$ intracellular levels and blocking phosphorylation of the myosin light-chain kinase.^187^ PBM causes vascular relaxation also via an increase of the activity of endothelial NO synthase (eNOS).^17^^,^^53^ The eNOS induces the NO production in the lymphatic endothelium that promotes an increase in lymphatic flow and removal of wastes and toxins (e.g., Aβ) from the brain and other tissues.^74^^,^^78^^,^^188^ Meanwhile, the increase in relaxation of MLVs mediated by tPBM also opens promising perspectives for lymphatic delivery of nanocarriers and drugs to the brain pathology bypassing the BBB. It was reported that a 1267 nm laser enhances the lymphatic transport of liposomes from the deep cervical lymph node (dcLN) to brain parenchyma and promotes the clearance of these nanocarriers from the subarachnoid space, offering a novel potential strategy for treating brain disease (Fig. 4).^182^ NO also controls lymphatic contractility, which is important for the movement of cells and molecules in the LVs.^189^ A hypothesis posits that a PBM-mediated release of NO could improve the lymphatic contractility, which might be another possible mechanism underlying PBM-activation of clearance of wastes and toxins from the brain (Fig. 4).^74^^,^^180^^,^^182^^,^^183^^,^^190^ Li et al. proposed that tPBM (1267 nm) reduced the mortality of an intraventricular hemorrhage (IVH) mouse model by promoting red blood cells (RBCs) transport from the ventricle to dcLNs. Significantly, they revealed that this beneficial effect disappeared when NO was blocked.^74^ In addition, it has been reported that sodium nitroprusside can increase the sensitivity of LVs to PBM,^191^ providing a potential strategy to further enhance the effects of tPBM treatment.

In additional to facilitating lymphatic drainage, the release of NO during tPBM could also increase cerebral blood flow^192^ and might contribute to the enhancement of cerebral endogenic and myogenic functional connectivity (FC).^171^^,^^193^^–^^195^

**Fig. 5:**
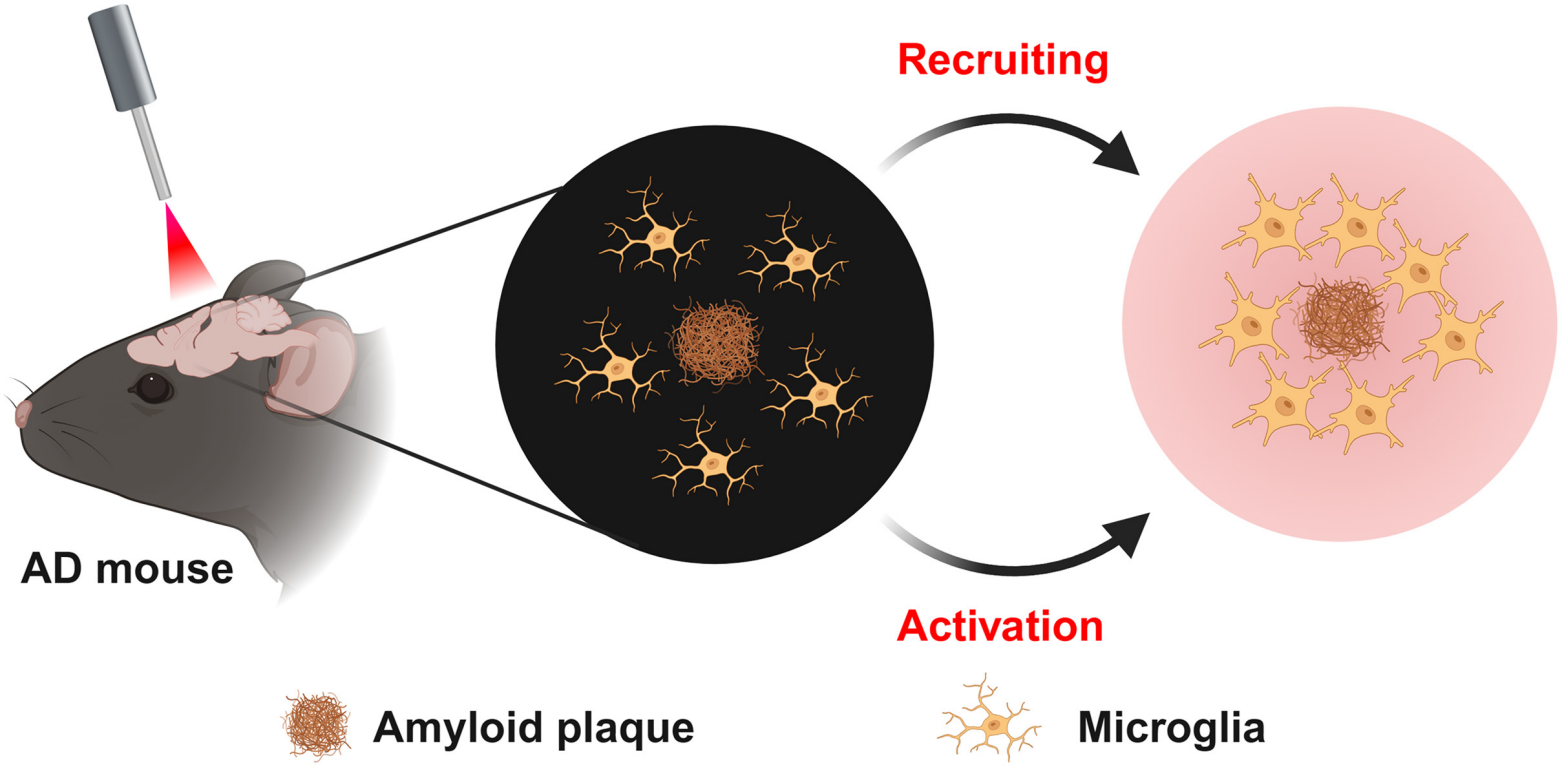
Illustration of a mechanism for tPBM treatment of AD: stimulating and recruiting microglia to the Aβ deposition.

Except for tPBM-mediated NO regulation, it has been demonstrated that tPBM can attenuate cerebral Aβ burden through the activation of the cAMP-dependent protein kinase signaling pathway, mediated by CCO, as well as via the stimulation of microglia and angiogenesis.^75^^,^^80^^,^^196^ In addition, Tao et al. reported that PBM (1070 nm) reduces the Aβ levels in the brain via stimulating and recruiting microglia to the Aβ burden^75^ (Fig. 5) and increasing cerebral vessel density.^75^^,^^196^ Yue et al. found that tPBM (630 nm) improves brain drainage leading to the Aβ removal in the APP/PS1 mouse model of AD.^81^

The release of the brain-derived neurotrophic factor (BDNF) may also contribute to PBM-mediated stimulation of Aβ elimination from the brain. Experiments *in vitro* indicated that PBM (632 nm) increases BDNF levels by activating the extracellular signal-regulated kinase/cyclic AMP-responsive-element-binding protein (CREB) pathway, which ameliorates Aβ-induced neuronal damage and dendritic atrophy.^197^ In addition, it has been demonstrated that tPBM (810 nm) significantly increases the BDNF level in the hippocampus of mice with brain trauma,^100^ depression,^120^ and ischemic stroke,^198^ which may be beneficial to neurogenesis and synaptogenesis.^199^

PBM with a light wavelength of 1267 nm can stimulate the generation of singlet oxygen directly in cultured cells and biological tissues without photosensitizers.^148^^,^^149^^,^^200^^,^^201^ Stanley et al. reported a new mechanism of singlet oxygen to regulate the endothelial relaxation via the control of the vascular tone.^150^^,^^152^ The singlet oxygen induces oxidation of the amino acid tryptophan in the mammalian tissues that leads cell production of metabolite, such as N-formylkynurenine with activation of the haem-containing enzyme called indoleamine 2,3-dioxygenase 1. This enzyme is widely expressed in the blood and lymphatic endothelium, contributing to the relaxation of vascular tone.^150^^–^^152^ Stanley et al. discovered that the endothelial indoleamine 2,3-dioxygenase 1 induced singlet oxygen-mediated relaxation of blood vessels.^150^ These pilot results provide new knowledge about singlet oxygen-related regulation of vascular tone and modulation of vascular responses to inflammation.

### 2.3 New Strategy in the Study of tPBM-Mechanisms

Glioblastoma (GBM) is the most common and aggressive form of brain cancer and is one of the deadliest brain tumors.^202^ Recently, it was discovered that GBM is characterized by reducing functions of the MLVs, which play an important role in the regulation of brain tumor drainage and immunity.^203^^–^^206^ These findings provide new strategies in the stimulation of an efficient immune response against glioma.^203^^,^^207^ Hu et al. revealed significant changes in the gene controlling the MLVs remodeling, fluid drainage, and inflammatory and immunological responses in mice with GBT.^203^ They discovered that mice with GBT and overexpression of the vascular endothelial growth factor C (VEGF-C) demonstrate a better response to anti-tumor therapy, such as the combination of anti-programmed death-1 protein (PD1) and cytotoxic T-lymphocyte-associated protein 4 (CTLA-4) blockade. It is interesting that the blockade of the MLVs abolished this effect.^203^ Indeed, mice with GBT treated with blockers of lymphatic proteins, such as chemokine (C-C motif) ligand 21 (CCL21) and C chemokine receptor type 7 (CCR7), did not show the improvement of anti-tumor therapy.^203^ Authors concluded that VEGF-C potentiates checkpoint therapy via the CCL21/CCR7 pathway. Song et al. also reported that VEGF receptor-3 (VEGFR3) enhances immune surveillance from GBM and improves the effectiveness of anti-tumor therapy with checkpoint inhibitors.^208^ Both Hu et al.^203^ and Song et al.^208^ documented an important role of the MLVs in the regulation of brain tumor immunity in mice. Because the tPBM-effects on the MLVs have already been documented,^17^^,^^74^ these pioneering results open new perspectives for the study of the role of tPBM in the stimulation of the CCL21/CCR7 pathway. This mechanism underlies the tPBM-induced effects on the MLVs and presents an alternative strategy for GBM treatment.

PBMs are a newly defined cell population. They reside in the leptomeninges and perivascular spaces along the vasculature and are responsible for regulating the cerebrospinal fluid flow dynamic. Researchers found that activating PBMs via intracisternal injection of the macrophage colony-stimulating factor could restore CSF dynamics in aging mice,^209^ which indicates that PBM activation via tPBM may help alleviate brain clearance deficits associated with aging and AD.

## 3 tPBM for Brain Diseases

### 3.1 tPBM for Ischemic Stroke

Ischemic stroke is a sudden cerebrovascular disease with high mortality. Some studies have proved that the BBB damage caused by ischemic stroke could increase the permeability of blood vessels, resulting in blood cells and neurotoxins entering the brain parenchyma and causing brain damage. It may activate inflammatory signals, trigger biochemical and molecular events, and further aggravate brain damage.^210^ In addition, ischemic stroke may alter the mitochondrial morphology and permeability, leading to the decrease of MMP and ATP levels, thereby inducing cell damage and apoptosis.^211^ Tissue plasminogen activator (TPA) is the only therapeutic approved by the U.S. Food and Drug Administration (FDA), but it must be received within 4.5 h after stroke for the effectiveness,^212^ and TPA may cause a more serious cerebral hemorrhage.

tPBM has the effects of mitochondrial function improvement and anti-inflammation, which is a potential therapeutic approach for ischemic stroke (see Table S1 in the Supplementary Material). In pre-clinical studies, Lee et al. found that transcranial LED light (610 nm) significantly reduced the ischemic infarct size, improved the neurological function score, inhibited neuroinflammation, and attenuated neuronal apoptosis in the photothrombotic mouse model.^54^ In addition, they also observed that applying the same tPBM protocol within 4 h after stroke improved long-term functional recovery and promoted neurogenesis and angiogenesis in a cerebral ischemic mouse model. This suggests that the therapeutic time window of certain tPBM was at least 4 h.^55^ Importantly, they also found that pretreatment with tPBM prevented the BBB damage and the blood flow decrease caused by ischemic brain injury.^51^^,^^53^ These beneficial effects were associated with the increase of expression of the TJ protein and the NO release in the blood endothelium. Recently, Kim et al. developed an implantable multi-LED (630 nm) array, which could prevent tissue and functional impairment, and attenuated the cognitive decline in an acute ischemic stroke mouse model.^52^ In addition, tPBM with wavelengths of 660,^56^ 780,^57^ 808,^58^^–^^60^ and 904^61^ nm also has beneficial effects of neuroprotection, anti-inflammation, and cognitive improvement in ischemic stroke rat models (including the middle cerebral artery occlusion model and the photothrombotic model). Oron et al. found that tPBM in the CW mode could better improve the neurological score and promote the neurogenesis in ischemic stroke mice than tPBM in the pulsed irradiation.^58^^,^^62^ Moreover, Lapchak et al. applied tPBM (808 nm) to the rabbit small clot electromagnetic stroke model to further verify the effectiveness. Specifically, they observed that applying tPBM with a power density of $7.5\;\mathrm{mW}/{\mathrm{cm}}^{2}$ within 3 h post-embolization significantly improved clinical rating scores. In addition, the therapeutic time window could be extended to 6 h by increasing the laser power density to $25\;\mathrm{mW}/{\mathrm{cm}}^{2}$.^63^ Similar dose-dependent beneficial effects were also observed by Huisa’s team.^64^ However, excessive power density may also lead to the plateau effect^65^ or even the biphasic effect.^66^ In addition, Lapchak et al. found that pulsed light (100 Hz) instead of continuous light could completely inhibit the decrease of ATP levels in the embolic cortex.^65^^,^^67^ Furthermore, it was reported that tPBM combined with TPA could better improve the behavior performance and mitigate the ATP decrease in the ischemic cortex.^68^^,^^69^

The clinical results of the NeuroThera Effectiveness and Safety Trial-1 (NEST-1) showed that tPBM (808 nm) within 24 h after stroke is safe and effective on clinical ischemic stroke treatment.^20^ However, the NEST-2 trial results showed that tPBM did not have a significant beneficial effect when the patient count and age distribution were increased.^21^ In addition, NEST-3 revealed that tPBM has no measurable neuroprotective effects in stroke patients.^70^ The above earlier studies may have had better results if more than one tPBM treatment had been applied and if tPBM had been applied to only the same side of the head, such as where the stroke had occurred. Naeser et al. reported that the naming ability in left-hemisphere (LH) stroke patients with lasting language problems (aphasia) was significant improved following 18 tPBM treatments applied to only one side of the head/scalp where the stroke had occurred. However, no improvement occurred when tPBM was applied to both sides of the head.^22^ In addition, a recent study demonstrated that the combination of tPBM and speech-language therapy resulted in greater improvements in speech-language skills post-stroke compared with speech-language therapy alone.^23^

### 3.2 tPBM for Hypoxic-Ischemic

An HI brain injury is a common clinical birth complication that has high mortality and disability rates.^213^ Currently, the only licensed therapeutic approach in clinic is hypothermia. However, it not only has a very narrow therapeutic time window but also has limited efficacy and usually comes with potential adverse cardiovascular effects.^214^ Therefore, there is an urgent need for a more practical and effective therapy for HI. It was reported that HI mainly damages mitochondria,^71^ causing mitochondrial structure (e.g., mitochondrial fragmentation) and function (e.g., reducing ATP production) impairment, thereby inducing oxidative stress and neuroinflammation. Therefore, protecting mitochondria from damage or enhancing mitochondrial function is a potential strategy for HI treatment.

Given that NIR light treatment has the potential to improve the mitochondrial function, Zhang’s laboratory explored whether tPBM (808 nm) has beneficial effects on neonatal HI (see Table S2 in the Supplementary Material). First, they found that applying tPBM immediately after HI for seven consecutive days contributed to robust neuroprotection effects by mitigating mitochondrial dysfunction, oxidative stress, and final neuronal apoptosis in rats.^72^ Second, they found that tPBM administered once 6 h before HI induction in postnatal 10 rats also mitigated brain damage.^73^ They also reported that applying tPBM three times per week to the abdomen of pregnant rats from gestation day (GD) 1 to GD 21 also exerted the neuroprotective effects against hypoxic ischemia in rat pups.^71^ In addition, Gerace et al. developed a dual-wavelength (808 and 904 nm) NIR laser source device that could attenuate the neurotoxicity of oxygen and glucose deprivation in hippocampal slices by attenuating inflammation.^215^ The above findings clearly show that tPBM might be a promising technology for therapy of HI in neonates. Further confirmation of animal data in clinical investigations will open a new niche of tPBM application in neonatology.

### 3.3 tPBM for Intracerebral Hemorrhage

IVH is defined as bleeding within the brain’s ventricular system, a condition associated with high rates of morbidity and mortality.^216^ Surgery and fibrinolysis in combination with extraventricular drainage is the conventional therapy of IVH. However, it has not made a significant impact on the natural history of IVH. Thus, new therapeutic approaches need to be urgently found to mitigate hematoma expansion and improve the drainage system of the brain.^217^ Li et al. demonstrated that an NIR tPBM (1267 nm) accelerates the RBCs evacuation from the ventricles to the dcLNs via the MLVs, leading to improvement of the neurological status in mice and newborn rat pups and their recovery after IVH (see Table S3 in the Supplementary Material).^74^

Li et al. optimized the tPBM illumination parameters for hemorrhagic stroke using the Monte Carlo method to stimulate photon propagation within the visible Chinese human head at a different level of intracerebral hemorrhage with varied parameters of light beams. They found that the Gaussian beam with a similar or larger size as the hemorrhagic region had the best therapeutic outcomes, whereas the top-hat beam performed better when the hemorrhagic region was much bigger than the beam size.^142^^,^^218^

### 3.4 tPBM for Alzheimer’s Disease

AD is a neurodegenerative disease that dramatically reduces the quality of life and ultimately leads to death.^219^ However, there is a lack of effective therapeutic strategies for AD in clinical practice. The amyloid cascade hypothesis proposes that the deposition of the Aβ peptide in the brain is a central event in disease pathology, which has long been the primary focus to develop therapeutic approaches that may slow or delay the progression of AD.^220^

Currently, there is compelling evidence suggesting that tPBM is capable of decreasing the Aβ burden and ameliorating cognitive and memory impairments in the AD animal models in various pathways (see Table S4 in the Supplementary Material). The latest trends suggest that tPBM (1267 nm),^79^ especially during deep sleep,^78^ stimulates lymphatic removal of Aβ from the mouse brain. A significant reduction of Aβ plaques in the hippocampus CA1 region was associated with improved recognition memory and cognitive status in mice (injection model of AD). This can be explained because night tPBM has a synergistic effect on the natural activation of mechanisms underlying night lymphatic drainage of brain parenchyma. Moreover, Xing’s lab proposed that tPBM (633 nm) shifted the amyloid precursor protein (APP) processing toward the nonamyloidogenic pathway by activating Sirtuin 1 via the cyclic adenosine monophosphate/protein kinase pathway, thereby improving memory and cognitive ability in an AD mouse model.^80^ They also showed that tPBM (633nm) could inhibit the activity of c-Jun N-terminal kinase 3 (a signal molecule related to neurodegeneration); this may be of therapeutic utility in the treatment of AD.^82^ Recently, Yue et al. provided new insight for AD treatment that showed that the use of a 630 nm laser in treatment could reverse Aβ-obstructed interstitial fluid flow and ameliorate memory decline in APP/PS1 mice.^81^ Grillo et al. observed 1072 nm laser treatment upregulated some stress response proteins in the AD mouse brain, known to reduce both Aβ aggregation and neuronal apoptosis.^83^ Tao et al. demonstrated that LED treatment at 1070 nm attenuated the Aβ burden by modulating the microglia phagocytosis capacity and promoting angiogenesis in AD mice.^75^ Zhang’s lab demonstrated that tPBM (808 nm) could not only mitigate Aβ-induced pathology (e.g., mitochondrial dysfunction, neuronal apoptosis, and tau pathology)^76^ but also prevent or slow the progression of AD in a rat model.^77^

In clinical trials, Berman et al. found that the application of tPBM (1060 to 1080 nm) for 28 consecutive days improved cognitive and memory ability in AD patients.^28^ In addition, three more recent tPBM clinical studies on mild to moderately severe dementia cases have been published; these included subjects who likely had a progressive neurodegenerative disease, e.g., AD, and tPBM was observed to improve cognition, mood, and sleep.^25^^–^^27^ Cases with MMSE entry scores of 10 to 24/30 and treated for 12 weeks showed significant cognitive improvements soon after the final tPBM treatment. However, 1 month later, there was a decline in their scores.^25^ Collectively, the above research with animal studies and early results from human clinical studies have put forward some tPBM mechanisms for mitigating AD, thus shedding light on a possible new treatment.

### 3.5 tPBM for Parkinson’s Disease

PD is a progressive neurodegenerative disorder, with more than 6 million patients worldwide. It is characterized by the loss of dopaminergic neurons in the substantia nigra and the presence of lewy bodies in the midbrain.^221^ Currently, the only available therapeutic approach for PD management is mainly based on exogenous dopaminergic supplements, such as levodopa. Such a treatment cannot modify the disease course or slow the underlying neurodegeneration associated with the multifactorial characteristics of PD.^222^ Some accepted points of view are that mitochondrial dysfunction, oxidative stress, and protein mishandling have a central role in PD pathogenesis.^223^ Furthermore, it has been reported that improving mitochondrial structure and function, thereby mitigating oxidative stress, could have beneficial effects for PD management.^224^ Consequently, tPBM has emerged as a potential therapeutic approach for PD. Based on this hypothesis, researchers have performed pre-clinical studies and clinical trials to explore whether tPBM could effectively treat PD. Some early results are provided in Table S5 in the Supplementary Material.

In pre-clinical trials, numerous studies demonstrated that tPBM (670 and 810 nm) effectively alleviated PD pathology in a 1-methyl-4-phenyl-1,2,3,6-tetrahydropyridine (MPTP)-induced PD mouse model. This included the prevention of the loss of tyrosine hydroxylase-positive (${\mathrm{TH}}^{+}$) cell and cerebrovascular leakage in the substantia nigra pars compacta (SNc) region.^84^^–^^90^ In addition, the neuroprotective effects of tPBM were also observed in lipopolysaccharide (LPS)-induced,^91^ 6-hydroxydopamine (6-OHDA)-induced,^92^ AAV2/6 virus-induced,^93^ and transgenic PD rat models^94^ and an MPTP-induced macaque monkey model.^95^ It should be noted that various factors may affect light penetration from the skull into the brain, thus affecting the therapeutic effects. Salgado et al. found that transcranial laser treatment, rather than LED, significantly reduced the level of serum pro-inflammatory cytokines.^92^ Mitrofanis’ group showed that the neuroprotective benefits of tPBM (670 nm) were more effective in the BALB/C strain than in the C57BL/6 strain. This could be attributed to deeper photon penetration, far into the brain from the skin in the BALB/C mice.^89^^,^^225^ They also discovered that the neuroprotective effects of tPBM existed in the SNc region but not in the periaqueductal grey matter (PaG) and zona incerta-hypothalamus (ZI-Hyp) regions. They attributed this to the fact that cell characteristics and photon penetration differ in different brain regions.^84^ Interestingly, Johnstone et al. observed that irradiation targeted to the dorsum and hindlimb also had neuroprotective effects, although not as effective as transcranial irradiation. This remote effect for PD treatment may be mediated by the regulation of variable signaling paths, such as stem cell-related C-X-C chemokine receptor type 4 signaling and oxidative stress response pathways.^96^^,^^226^

In clinical trials, McGee et al. found that tPBM (635 nm plus 810 nm LEDs) could safely and meaningfully improve individual motor signs of PD.^33^ Hamilton et al. developed a helmet device with several LEDs with wavelengths of 670, 810, and 850 nm and reported improvement in PD progression.^31^ In fact, owing to the exponential attenuation of light traveling through skull and brain tissues, only a very limited number of neurons can absorb sufficient photons for positive effects. Therefore, various PBM strategies were developed for clinical PD treatment. Liebert et al. stressed the importance and demonstrated the effectiveness of treating the abdomen and the brain with PBM (gut/brain axis) in PD.^30^ Other light delivery strategies, such as neck and intranasal PBM, were also used in combination with tPBM for clinical PD treatment and reported effectivenss.^29^^,^^34^ Hong et al. found that tPBM (940 nm) targeting the brainstem combined with molecular hydrogen treatment significantly reduced the Unified Parkinson Disease Rating Scale scores in PD patients. Such an improvement still existed in the follow-up period of 1 week after treatment.^32^ In summary, the above encouraging results of pre-clinical studies and clinical trials show the great potential of tPBM in PD treatment.

### 3.6 tPBM for Multiple Sclerosis

MS is one of the most common chronic inflammatory, demyelinating, and neurodegenerative diseases of the CNS in adults; it affects two to three million people worldwide.^227^ One of the prominent characteristics of MS is the infiltration of human type 1 helper lymphocytes from peripheral blood into the brain and the spinal cord, leading to the activation of microglia, which induces neuroinflammation, thus causing neuronal death and demyelination.^228^ Currently, there is no effective therapeutic approach for MS; this motivates researchers to seek alternative treatment for brain function recovery. tPBM has attracted attention for MS due to its effectiveness on the regulation of inflammation and the promotion of neurogenesis.^97^

In pre-clinical studies, Duarte et al. found that tPBM improved motor performance in mice with demyelination. This improvement may be associated with the attenuation of demyelination, proliferation of oligodendrocyte precursor cells, and inhibition of neuroinflammation.^98^ In clinical trials, Silva et al. found that tPBM effectively upregulated the expression level of interleukin-10 in MS patients.^97^ They also found that tPBM improved the fatigue status of MS patients, despite no statistical significance in the modified fatigue impact scale measurement.^99^ These studies (see Table S6 in the Supplementary Material) have suggested evidence for support of tPBM as a possible treatment for MS. Future investigations are likely to provide positive results.

### 3.7 tPBM for Traumatic Brain Injury

TBI is defined as an alteration in brain function or other evidence of brain pathology caused by an external trauma.^229^ The pathophysiology of TBI is highly heterogeneous and complex, including adverse signaling pathways activation, inflammation, oxidative stress, mitochondrial dysfunction, and excitotoxic damage. The combination of cellular and physiological disturbances increases the infarct size, neurological decline, and cognitive impairment. TBI occurs more than 50 million times annually worldwide, posing a significant burden on socio-economic and healthcare systems.^230^ Currently, due to the heterogeneity of TBI and limited understanding of potential pathophysiological mechanisms, there are no standardized methods or drug treatments.^231^ This has led to interest in new treatment approaches such as tPBM. Since the 21st century, many research teams have begun to explore the potential of tPBM as a treatment for acute or chronic TBI. There have been many positive results (see Table S7 in the Supplementary Material).

Hamblin’s team found that one transcranial, red (660 nm), or NIR (810 nm) laser treatment performed at 4 h after TBI significantly improved the neurological severity score (NSS) and decreased the brain lesion volume in moderate-to-severe TBI mouse models.^101^^,^^102^ They also reported that tPBM, when applied during three consecutive days post-TBI, was more effective than only one irradiation, in improving the motor and memory ability in the TBI mouse model.^103^ This reduced the degeneration and apoptosis of neurons in the injured region,^103^^,^^104^ promoting the neurogenesis, synapse formation, and expression level of BDNF in the dentate gyrus (DG) of the hippocampus and in the subventricular zone.^104^^,^^105^ However, it was also reported that an excessive number of tPBM treatments in mice with TBI could temporarily inhibit the process of brain repair, suggesting that it is important to choose the optimal protocol of tPBM for TBI.^106^ Shemesh et al. found that tPBM (810 nm) regulated the hemodynamics, with reduced cell death and stimulation of neurogenesis.^107^ Wu’s lab found that tPBM (810 nm), combined with energy metabolism regulators (e.g., lactic acid or pyruvate), more effectively increased ATP levels in the impact cortex and reduced neuronal damage and neuroinflammation caused by the TBI.^108^^,^^109^ Micci’s team found that tPBM combined with ultrasound (optoacoustic) treatment effectively mitigated sympathetic dysfunction, neuroinflammation, and dysregulation of neurogenesis in the blast brain injury mouse model.^100^^,^^110^ Similarly, neuroprotective effects of tPBM for TBI in pre-clinical studies were observed in the laboratories of Whalan,^111^^,^^112^ Marques,^113^ Oron,^114^^,^^115^ and Zhang.^116^

Some of the above studies compared therapeutic effects from different light parameters. For various wavelengths, Reinhart et al. found that tPBM at 660 and 810 nm, but not at 730 or 980 nm, had neuroprotective effects.^102^ This can be due to weak light absorption by CCO of 730 and 980 nm.^102^ Regarding the delivery mode, Oron et al. found that the 100-Hz pulsed laser improved the NSS better than CW and the 600-Hz pulsed laser in a TBI mouse model. The authors speculated that this phenomenon may be related to the resonance effect between 100-Hz PW laser and brain waves (such as alpha and theta waves).^115^ Also, Ando et al. proposed that a 10-Hz pulsed laser has more neuroprotective and cognitive improvement effects than a 100-Hz PW laser. This may be induced by a positive resonance between the 10-Hz PW laser and the electrical activity of neurons in the hippocampus.^101^ Both Abookasis and Whalen’s team found that the therapeutic effects of one tPBM treatment was positively correlated with the energy density within a specific range.^107^^,^^112^

In clinical trials, Naeser et al. found that tPBM (at 633 and 870 nm) treatment significantly improved the cognitive performance in mild TBI patients.^36^ The team also reported that tPBM could enhance sleep duration by an average of 1 h in chronic TBI cases,^37^^,^^38^ for which poor sleep is a common complaint. Longo et al. studied acute, hospitalized, moderate TBI cases with magnetic resonance imaging (MRI) and reported significant differences between MRI-derived diffusion parameters in white matter tracts between sham versus real LED-treated groups, demonstrating safety and neuro-reactivity for real tPBM in this population.^39^ Nawashiro et al. used single-photon emission computed tomography brain scans, after 73 days of LED (850 nm) tPBM, applied twice a day to the left and the right forehead areas and reported that the regional CBF (rCBF) increased by 20% in the left anterior frontal lobe in a severe TBI patient who was in a persistent vegetative state.^40^ This was associated with some new arm movement. In addition, Chao et al. reported that after combining intranasal plus transcranial PBM (810 nm) treatments for 8 weeks, there was increased brain volume, improved FC, increased cerebral perfusion, and improved neuropsychological test scores in an athlete, age 23, who had had six concussions in 5.5 years.^35^

In conclusion, tPBM for TBI mitigates the death of brain neurons, decreases neuroinflammation, and improves the self-repair ability of the brain by stimulating synapses formation and proliferation of nerve cells, demonstrating high potential of tPBM for the clinical treatment of TBI.

### 3.8 tPBM for Possible Chronic Traumatic Encephalopathy

CTE is a progressive neurodegenerative disease present in athletes who have sustained repetitive head impacts. At post-mortem, hyper-phosphorylated tau deposits (p-tau) are present in the deep sulcal areas, which is unique to CTE. It differs from AD, in which Aβ and another form of tau deposits are present in different areas of the brain. Symptoms of CTE include behavioral and mood changes, memory loss, cognitive impairment, and dementia.^232^ However, there are no known current treatments for CTE.^233^ Recently, Naeser et al. reported the new use of tPBM to treat possible CTE.^117^ This study showed significant improvements in cognition and behavior/mood for four ex-football players after 18 tPBM treatments (see Table S8 in the Supplementary Material). At 2 months after this first in-office, tPBM treatment series was completed, two of these ex-football players regressed. Then, home tPBM treatments were self-applied to only cortical node areas of the default mode network (12 weeks). Again, significant improvements returned. Increased FC for the salience network, post-tPBM, was present. Also, increased n-acetyl-aspartate (NAA), reflecting increased oxygen consumption in the mitochondria of neurons, was present in the anterior cingulate cortex, parallel to less pain and PTSD, and lasted for 12 weeks post-tPBM. Ongoing, tPBM treatments that can be safely applied at home may be necessary to maintain gains in cases with progressive neurodegenerative disease.

### 3.9 tPBM for Depression

Depressive disorder is one of the most prevalent and debilitating forms of psychopathology;^234^ it manifests by lack of energy, depressed mood, low executive ability, and poor concentration. Epidemiological surveys indicate that depressive disorder affects more than 16% of the worldwide population. This increases the prevalence of medical illnesses, such as type 2 diabetes, cardiovascular diseases, and autoimmune diseases, and it even advances biological aging.^235^ At present, pharmaceutical intervention is the most mainstream therapeutic approach for depression, but it has a low effective rate and even some opposite reactions. Electroconvulsive therapy (ECT), TMS, and vague nerve stimulation are FDA-approved neuromodulation strategies for the treatment of depression. However, complex procedures, such as anesthesia for ECT, as well as expensive costs limit their clinical application.^236^ Therefore, there is urgent need for a safe, effective, and convenient treatment strategy for depression that may be well tolerated and potentially used by patients at home. It has been reported that mitochondrial dysfunction, cerebral energy metabolism impairment, and oxidative stress may play a significant role in the development of depression.^237^ tPBM has been reported to modulate a variety of biological processes, including anti-oxidation, anti-inflammation, neuro-enhancement, ATP synthesis, etc.^238^ This suggests that tPBM may be an attractive, new treatment for depression. Currently, extensive pre-clinical studies and clinical trials have demonstrated that tPBM indeed has beneficial effects on depression (see Table S9 in the Supplementary Material).

Salehpour et al. found that tPBM (810 nm) significantly improved the neurological status in sleep-deprived and, in restraint, stress-induced depression mouse models. The authors proposed that reversing oxidative stress, neuroinflammation, and neuronal apoptosis in the prefrontal cortex (PFC) and hippocampus might be a target for the beneficial effects of tPBM in these models.^118^^,^^119^ Xu et al. found that tPBM (808 nm) elevated PFC ATP levels and mitochondrial complex IV activity in space restriction and abelson helper integration site-1 (Ahi1) knockout (KO) depression mouse models.^121^ Farazi found that tPBM (810 nm) attenuated the decrease of BDNF, tropomyosin receptor kinase B (TrkB), and phospho-CREB/CREB in the hippocampus and down-regulated the serum corticosterone levels in mice with noise-induced depression.^120^ tPBM also promoted neuroprotection effects from stress,^122^^,^^123^ reserpine,^124^ underwater trauma,^125^ and early AD-associated^126^ depression in rat models. Specifically, Tanaka et al. found that tPBM (bright light) significantly promoted neurogenesis in the hippocampal CA1 region.^239^ Li et al. found that tPBM (810 nm) elevated ATP levels in the hippocampus in rats with a post-traumatic stress disorders model and modulated activated neurons expressing immediate-early genes, such as Arc and c-fos in the hippocampus and amygdala.^125^ Mohammed et al. found that low-dose (80 mW) tPBM significantly improved depression-like behavior in reserpine-induced depression in rats. Using electro-corticography spectral analysis, the beneficial effects of tPBM (80 mW) also included the inhibition of abnormal elevation of the Delta frequency band and decline of the Beta-1 and Beta-2 bands.^124^ Salehpour et al. observed that a 10-Hz pulsed NIR laser (808 nm) was as effective as the Citalopram treatment and was more effective than a red laser (660 nm) in improving depressive-like behaviors in rats. This was likely due to a better penetration depth and higher absorption by CCO of the 808 nm laser.^123^

In clinical trials, Schiffer et al. found that tPBM (810 nm) significantly improved the Hamilton depression rating scale and Hamilton anxiety rating scale scores in depression patients. There was increased mean rCBF in both hemispheres.^42^ Cassano et al. confirmed the safety and effectiveness of tPBM (823 nm) in major depressive disorder patients.^43^ In addition, Disner et al. found that applying tPBM to the right prefrontal lobe enhanced the therapeutic effects of attention bias modification.^41^ Kerppers et al. found that tPBM (945 nm) was also effective for the treatment of depression.^44^ In conclusion, the above compelling evidence shows that tPBM could be a promising alternative method for the therapy of depression in routine clinical practice.

### 3.10 tPBM for Aging

Aging is a multifactorial biological process, manifested by a progressive functional decline at the molecular, cellular, tissue, and organ levels. In addition, aging often increases the susceptibility to neurodegenerative and cardiovascular diseases, diabetes, and even cancer.^240^ Epidemiological studies show that 11% of the world population was over 60 years old in 2016, and it was estimated that this proportion will increase to 22% by 2050.^241^ Although people welcome the prospect of longer life, they hope that old age is healthy rather than accompanied by disease. Therefore, it is important to explore the physiological and pathological processes of aging and find effective anti-aging therapeutics in the increasingly aging society. Oxidative stress and mitochondrial dysfunction are two important factors associated with aging.^242^ Specifically, the oxidative stress theory indicates that the imbalance between the production of pro-oxidants and the defense of anti-oxidants during aging leads to excessive production and accumulation of ROS, damages macromolecules (lipids, DNA, and proteins), and then induces cell aging and death.^243^ In addition, mitochondria are considered to be the main target of oxidative damage, the dysfunction of which during aging is associated with cognitive impairment.^244^ Furthermore, oxidative stress within mitochondria can lead to a vicious cycle in which damaged mitochondria produce increased amounts of ROS, leading in turn to progressive augmentation in damage. Therefore, improving mitochondria function and inhibiting oxidative stress may have beneficial effects on the pathology associated with aging (see Table S10 in the Supplementary Material).

In pre-clinical studies, Sadigh–Eteghad’s team found that transcranial red (660 nm) and NIR (810 nm) light treatment significantly alleviated memory and cognitive decline, mitochondrial dysfunction, oxidative stress, and cell apoptosis in the D-galactose-induced aging mouse model.^127^^–^^129^ Importantly, they found that a medium dose ($8\;J/{\mathrm{cm}}^{2}$ per day) was more effective than a low dose ($4\;J/{\mathrm{cm}}^{2}$ per day) or a high dose ($32\;J/{\mathrm{cm}}^{2}$ per day). There was no significant difference between red light (660 nm) and NIR light (810 nm). They also observed that red light (660 nm) alleviated cognitive impairment and mitochondrial dysfunction in naturally aging mice (18 months old).^130^ Moreover, the cognitive improvement effect was also observed by 1072 nm laser treatment in Ennaceur’s team.^131^ Massri et al. found that tPBM (670 nm) had beneficial effects on the inhibition of age-related glial cell (e.g., astrocyte and microglia) proliferation.^132^ Cardoso’s team found that aging primarily leads to a decrease of regional brain CCO activity and impairment of systems-level FC and that tPBM (810 nm) reversed these age-related effects.^133^ Furthermore, they also observed that tPBM mitigated the inflammatory response and altered the intracellular signaling pathways linked to vascular function, cell survival, and glucose metabolism in the aged brain.^134^^–^^136^

In clinical trials, Salgado et al. found in elderly women that, using transcranial Doppler ultrasound measurements, tPBM (627 nm) significantly increased the systolic and diastolic velocities of the left middle cerebral artery and the basilar artery and decreased the fluctuation index and resistance index.^46^ In addition, Chan et al. demonstrated improved depressive state and cognitive function of the elderly after applying combined red (633 nm) and NIR (870 nm) LEDs.^48^^,^^49^ Moreover, Saucedo et al. found that the beneficial effects of tPBM (1064 nm) for the elderly were not limited to improving cognitive function but also included increasing resting-stateEEG alpha, beta, gamma power, and prefrontal blood-oxygen-level-dependent -fMRI activity.^45^ They also found that the CCO level was elevated during the irradiation period, followed by a significant post-stimulation increase in oxygenated hemoglobin and a decrease in deoxygenated hemoglobin.^47^ Qu et al. observed that 7-day repeated tPBM efficiently improved the working memory of healthy older adults, with the beneficial effects lasting at least 3 weeks.^50^ The above results indicated that tPBM could be a promising candidate for age-related cognitive improvement. In the future, there will be an urgent need to explore the functional changes related to phototherapy on the aging brain, including molecular and electrophysiological measurements and optical imaging techniques.

### 3.11 tPBM for Epilepsy

Epilepsy is a lifelong condition characterized by spontaneous and recurrent seizures. Epilepsy affects 70 million people worldwide and entails a major burden in seizure-related disability, mortality, comorbidities, stigma, and costs.^236^ Currently, anti-seizure medicines are the prevailing treatment modality for most people with epilepsy and are usually accompanied by low response rates (∼70%) and high recurrence rates (>50%).^137^ Although invasive neuronal stimulation treatments, such as TMS and tDCS, have been established as palliative treatments for patients with drug-resistant epilepsy, the low effectiveness and the high adverse event rate limit their clinical application.^137^ Therefore, an effective and non-invasive therapeutic for epilepsy is urgently needed. Recently, tPBM with NIR light has been proposed as a new alternative treatment for epilepsy in animal model experiments (see Table S11 in the Supplementary Material).

Radwan et al. observed that tPBM (830 nm) reverses pilocarpine induced neurochemical changes of amino acid neurotransmitters (e.g., increase of glutamate), and they found that such benefits could be due to the regulation of transaminase activity by laser irradiation.^138^ Vogel et al. observed that tPBM (780 nm) reduces epileptiform discharges in post-stroke epilepsy rats. The authors reasoned that the beneficial effects of tPBM might be responsible for the disruption and mitigation of self-regulatory mechanisms and complex network reorganization.^139^ Tsai et al. found that applying tPBM before inducing epilepsy attenuated pentylenetetrazole (PTZ)-induced severe seizures, status epileptics, and mortality in peripubertal rats. This could be explained by protecting hippocampal parvalbumin-positive interneurons from apoptosis and preserving the integrity of the parvalbumin-positive perisomatic inhibitory network.^137^ They also found that tPBM (808 nm) mitigated neuroinflammation and glial cell activation in the hippocampus in a CCO-dependent manner in the PTZ-induced epilepsy rat model.^140^ Hong et al. observed that tPBM could prevent seizure-induced neuronal degeneration.^141^ In summary, the above studies demonstrated that tPBM with NIR light could be potential non-invasive and effective therapeutics for inhibiting epileptogenesis and alleviating epilepsy damage.

## 4 Conclusion

In this review, we discussed new discoveries of therapeutic effects of tPBM to shed light on tPBM-mediated mechanisms of treatment for brain diseases. We also highlighted emerging trends in tPBM application for modulation of the immune system of the brain and stimulation of the MLVs, thus opening a new niche in tPBM-immunotherapy of brain diseases and in the removal of waste and toxins from the CNS. We summarized the animal experiments and clinical trials in different brain diseases. This is expected to provide guidance for future experimental designs and clinical applications.

Among the main disadvantages of tPBM is the limited information about the optimal tPBM protocols for the effective therapy of specific brain diseases. Despite the large number of experimental and clinical studies, it remains unclear whether the tPBM effects on the energy, metabolic, hemodynamic, drainage, and immune processes of the brain are nonspecific or whether they depend on specific characteristics of brain diseases. A better understanding of the dose-dependent effects of tPBM on the brain physiology and mechanisms of tPBM therapy will help to develop the optimal and personalized tPBM standards and guidelines of this therapy for brain diseases.

The light delivery strategy of tPBM is also important for the treatment of brain diseases. The majority of the light delivery approaches are transcranial or intranasal. However, photons are scattered by the scalp, skull, meninges, and cerebrospinal fluid. Thus, only a small number of photons can effectively the reach brain tissues. In fact, in most brain diseases, deeper areas of the CNS are also involved. For example, in patients with PD, the midbrain is characterized by the progressive, selective loss of dopaminergic neurons in the SNc region. Therefore, the development of innovative strategies to deliver light deeper, including, for example, modern lasers (e.g., 1267 nm) with deep penetration into brain tissues, will open a new era in tPBM for treatment of brain diseases.

Given that the BBB prevents drug delivery to the brain and significantly limits the advancement in the development of new pharmacological therapies for brain diseases, tPBM can be an important therapeutic approach for preventing or delaying neurological pathologies. It is important to note that some companies have ceased initiatives to finance the development of pharmacological treatments for AD. Therefore, tPBM-mediated stimulation of the MLVs and removal of toxins, including Aβ from the brain, might be an important method for the treatment of this disease. tPBM-stimulating effects on the mechanisms of lymphatic drainage and the brain immune system open pioneering strategy for neuroprotection of the CNS in various brain diseases.

The special advantages of tPBM include its safety, ease of use in clinical settings and at home, and cost effectiveness. This makes tPBM attractive in the market of medical devices. Therefore, a better understanding of the mechanisms of tPBM, the development of generally accepted guidelines for tPBM use in clinical practice, and the study of dose-dependent and personal tPBM needs/effects hold great promise for progress in the treatment of brain diseases.

## Data Availability

Data sharing is not applicable to this article, as no new data were created or analyzed.
