# Supplementary material for: Transcranial photobiomodulation for brain diseases: review of animal and human studies including mechanisms and emerging trends
Source: Neurophotonics. 2024 Feb 5;11(1):010601. doi: 10.1117/1.NPh.11.1.010601 (PMC10840571; doi:10.1117/1.NPh.11.1.010601)
Supplement: Supplementary file 1 [file NPh_011_010601_SD001.pdf]

Supplementary Material

Transcranial photobiomodulation for brain diseases: Review of animal and human studies including mechanisms and emerging trends

Hao Lin,[a,†] Dongyu Li,[a,b,†] Jingtan Zhu,[a] Shaojun Liu,[a] Jingting Li,[c] Tingting Yu,[a] Valery V. Tuchin,[d,e,f] Oxana Semyachkina-Glushkovskaya,[d,g,*] Dan Zhu[a,*]

[a]Huazhong University of Science and Technology, Wuhan National Laboratory for Optoelectronics - Advanced Biomedical Imaging Facility, Britton Chance Center for Biomedical Photonics, Wuhan, Hubei 430074, China

[b]Huazhong University of Science and Technology, School of Optical Electronic Information, Wuhan, Hubei 430074, China

[c]Huazhong University of Science and Technology, School of Engineering Sciences, Wuhan, Hubei 430074, China

[d]Saratov State University, Science Medical Center, Astrakhanskaya str 83, Saratov 410012, Russia

[e]Research Center of Biotechnology of the Russian Academy of Sciences, Bach Institute of Biochemistry, Moscow 119071, Russia

[f]Tomsk State University, Laboratory of Laser Molecular Imaging and Machine Learning, Tomsk 634050, Russia

[g]Humboldt University, Physics Department, Newtonstrasse 15, Berlin 12489, Germany

Tables S1 – S11

Table S1. Reports of tPBM used for ischemic stroke in pre-clinical and clinical studies.

Authors/ year	Animal/ species	Pathological model	Parameters of the light used	Protocol	Effects
Lee et al. [51] (2016)	Male C57BL/6J mouse	Photothrombotic model	LED; 610 nm; 20 min 1.7 mW/cm^2 on the scalp Spot diameter: 4 mm 2.0 J/cm^2 on the scalp	Twice a day for 2 days prior to stroke	1. Reduced infarct size and edema, and improved neurological scores. 2. Inhibited gliosis and decreased the level of pro-inflammatory cytokines. 3. Inhibited mitogen-activated protein kinase activation and nuclear factor-κB translocation. 4. Prevented the destruction of BBB.
Lee et al. [54] (2017)	Male C57BL/6J mouse	Photothrombotic model	LED; 610 nm; 20 min 1.7 mW/cm^2 on the scalp Spot diameter: 4 mm 2.0 J/cm^2 on the scalp	Twice a day for 2 days prior to stroke	1. Inhibited the infiltration of neutrophils. 2. Decreased the number of TUNEL$^+$/PI$^+$ cells and the expression of cleaved Caspase-1 and Caspase-11. 3. Inhibited the expression of NLRP3 inflammatory body.
Lee et al. [55] (2017)	Male C57BL/6J mouse	Photothrombotic model	LED; 610 nm; 20 min 1.7 mW/cm^2 on the scalp Spot diameter: 4 mm 2.0 J/cm^2 on the scalp	Once a day for 7 days immediately (acute treatment group) or from forth day (subacute treatment group) after stroke	1. Improved motor function 28 days after ischemic stroke in both treatment groups. 2. tPBM (subacute treatment) increased BrdU$^+$/GFAP$^+$ and decreased BrdU$^+$/Iba-1$^+$ expression in the cortex. 3. tPBM (subacute treatment) promoted the proliferation and differentiation of neuronal cells. 4. tPBM with subacute treatment upregulated BDNF level.
Lee et al. [53] (2017)	Male C57BL/6J mouse and Male eNOS$^{-/-}$ mouse	Middle cerebral artery occlusion/reperfusion (MCAO/R)	LED; 610 nm; 20 min 1.7 mW/cm^2 on the scalp Spot diameter: 4 mm 2.0 J/cm^2 on the scalp	Twice a day for 2 days prior to stroke	1. Pretreatment reduced infarct size and edema, and improved neurological scores and motor function. 2. Partially recovered cerebral blood flow after reperfusion. 3. Activated PI3K/Akt pathway, upregulated eNOS phosphorylation, and promoted the generation of NO.
Kim et al. [52] (2022)	Male C57BL/6J mouse	Photothrombotic model	LED; 630 or 850 or 940 nm; 20 min; 17 mW/cm^2 on the scalp	Twice a day for 3 days before stroke (scheme 1) or twice a day for 3 days after stroke (scheme 2)	1. tPBM (630 nm) with scheme 1 reduced cortex infarct size, recovered behavioral function, increased the expression of CD31$^+$ and decreased the expression of Iba-1$^+$ and GFAP$^+$. 2. tPBM (630 nm) attenuated AIM2 inflammasome activation and inflammasome-mediated pyroptosis.
Leung et al. [56] (2002)	Male Sprague-Dawley rat	Middle cerebral artery occlusion (MCAO)	Laser; 660 nm 1 or 5 or 10 min Spot size: 20 mm^2 44 mW/cm^2 2.64 or 13.2 or 26.4 J/cm^2 PW mode: 10 kHz	Single treatment immediately after stroke	1. Reduced the activity of NOS and its subtypes (i.e. eNOS, iNOS and nNOS) with dose dependency. 2. Triggered the expression of TGF-b1.
Oron et al. [58] (2006)	Male Sprague-Dawley rat	MCAO	Laser; 808 nm 7.5 mW/cm^2 on the cortex 0.9 J/cm^2 on the cortex PW (70 Hz) or CW	Single treatment from 4 hours (scheme 1) or 24h (scheme 2) after stroke	1. tPBM with scheme 2 improved neurological scores. 2. tPBM (scheme 1) with PW mode had a better motor improvement effect than CW mode treatment 28 days after stroke. 3. tPBM with CW mode had a better neurogenesis effect than PW mode treatment.
DeTaboada et al. [62] (2006)	Sprague-Dawley rat	MCAO	Laser; 808 nm 7.5 mW/cm^2 on the cortex 0.9 J/cm^2 on the cortex	Single treatment at ipsilateral (scheme 1) or contralateral (scheme 2) or both sides (scheme 3) of ischemic region	1. tPBM, especially scheme 2, improved modified neurological scores.
Yang et al. [60] (2018)	Male Sprague-Dawley rat	Photothrombotic model	LED; 808 nm; 2 min	Once a day for 7 days from 24	1. Improved motor ability. 2. Reduced infarct area and increased the number of surviving neurons in the cortex.

			350 mW/cm^2 on the scalp<br>25 mW/cm^2 on the cortex	hours after stroke	3. Inhibited the damage of dendrites and synapses.<br>4. Increased the expression level of stem cell proliferation markers (e.g. Ki67) and immature neuron markers (e.g. DCX) in the infarction region.<br>5. Increased CCO activity and ATP levels.<br>6. Decreased the expression level of proinflammatory cytokines (e.g. TNF-α) and promoted the transformation of microglia phenotype from M1 to M2.
Wang et al. [59] (2019)	Male Sprague-Dawley rat	Global cerebral ischemia induced by embolic bilateral common carotid arteries	Laser; 808 nm<br>Spot size: 1 cm^2<br>8 mW/cm^2 on the hippocampus<br>1 or 2 or 3 or 4 J	Once a day for 14 days	1. tPBM with dose of 3 or 4 J mitigated neuron death in hippocampal CA1 region.<br>2. Attenuated hippocampal-dependent short-term (1 week) and long-term (6 months) behavioral deficits.<br>3. Preserved mitochondrial dynamics and suppressed substantial mitochondrial fragmentation of hippocampal CA1 neurons.<br>4. Reduced mitochondrial oxidative damage, restored mitochondrial overall health status, and preserved mitochondrial function.
Fonseca et al. [61] (2019)	Wistar rat	Electrolytic damage of electrode induced	LED; 904 nm; 63 s<br>7 J/cm^2	Once a day for 7 days (scheme 1) or 21 days (scheme 2)	1. tPBM with scheme 2 improved muscle resistance.<br>2. tPBM with both schemes mitigated anxiety condition.<br>3. tPBM with both schemes promoted neurogenesis.
Vogel et al. [57] (2021)	Male Wistar rat	Photothrombotic model	LED; 780 nm; 2 min<br>0.083 W/cm^2 on the scalp<br>10 J/cm^2 on the scalp	3 times per week for 60 days from 24 hours after stroke	1. Increased the number of GFAP$^+$ cells and decreased the number of Iba-1$^+$ cells.<br>2. Reduced the expression level of proinflammatory cytokines TNF-α, IL-1β and IL-6.<br>3. No further positive effects combined with anti-inflammatory medicine Ω-3 treatment.
Lapchak et al. [63] (2004)	Rabbit	Rabbit small clot embolic stroke model (RSCEM)	Laser; 808 nm<br>Dose 1:<br>7.5 mW/cm^2 on the cortex<br>0.9 J/cm^2 on the cortex<br>Dose 2:<br>25 mW/cm^2 on the cortex<br>15 J/cm^2 on the cortex	Single treatment from 1 (scheme 1) or 3 (scheme 2) or 6 (scheme 3) hours after stroke	1. tPBM (scheme 1 and 2) with low dose (dose 1) more efficiently mitigated motor impairment.<br>2. tPBM (scheme 3) with high dose (dose 2) more efficiently mitigated motor impairment.
Lapchak et al. [67] (2007)	Male new zealand white rabbit	RSCEM	Laser; 808 nm; 2 min<br>Spot diameter: 2 cm<br>7.5 mW/cm^2 on the cortex<br>CW or PW 1 (1 kHz, 30%) or PW 2 (100 Hz, 20%)	Single treatment from 6 hours after stroke (scheme 1) or single treatment from 12 hours after stroke (scheme 2)	1. tPBM (PW 1 and 2 modes) improved clinical rating scores.
Lapchak et al. [68] (2008)	Male new zealand white rabbit	RSCEM	Laser; 808 nm; 2 min<br>10 mW/cm^2 on the cortex<br>1.2 J/cm^2 on the cortex	Single treatment from 90 min after stroke	1. tPBM could be combined with tPA for stroke treatment.<br>2. No abnormal mortality and bleeding were observed.
Lapchak et al. [65] (2010)	Male new zealand white rabbit	RSCEM	Laser; 808 nm; 2 min<br>CW: 7.5 mW/cm^2 on the cortex<br>PW 1: 37.5 mW/cm^2 on the cortex<br>100 Hz<br>PW 2: 262.5 mW/cm^2 on the cortex	Single treatment from 5 min after stroke	1. tPBM with PW mode (especially PW 2) more effectively rescued ATP level in the cortex than CW mode.

			100 Hz		
Huisa et al. [64] (2013)	Male new zealand white rabbit	RSCEM	Laser; 808 nm; 2 min	Single (scheme 1) or double (scheme 2) or triple (scheme 3) treatments after stroke	1. Improved neurological function scores with treatment times dependency.
Lapchak et al. [69] (2016)	Male new zealand white rabbit	RSCEM	Laser; 808 nm<br>7.5 mW/cm^2 on the cortex<br>0.9 J/cm^2 on the cortex	Single treatment from 1 hour after stroke	1. Improved motor function and increased ATP levels in ischemic cortex.<br>2. Had a better therapeutic effect combined with tPA treatment.
Meyer et al. [66] (2016)	Male new zealand white rabbit	RSCEM	Laser; 808 nm; 2 min<br>CW: 7.5 or 10.8 or 20 or 55.6 mW/cm^2 on the cortex<br>PW: 55.6 or 111 or 333 mW/cm^2 on the cortex<br>10 or 100Hz	Single (scheme 1) or triple (scheme 2) treatment from 2 hours after stroke	1. 100 Hz-laser treatment with dose of 111 mW/cm^2 was safe, and had best motor improvement effect.<br>2. Exists biphasic effect.
Lampl et al. [20] (2007)	Human	–	Laser; 808 nm; 6 min<br>1 J/cm^2 on the cortex	Single treatment at 20 predetermined sites within 24h of stroke	1. Improved Bnih measure score, mRs score, Barthel index and Glasgow outcome scale.<br>2. Had no adverse effect on mortality and serious adverse event.
Zivin et al. [21] (2009)	Human	–	Laser; 808 nm; 2 min	Single treatment at 20 predetermined sites within 24h of stroke	1. Not significantly improved modified Rankin Scale score and National Institutes of Health Stroke Scale score.
Hacke et al. [70] (2014)	Human	–	Laser; 808 nm; 2 min	Single treatment at 20 predetermined sites within 24h of stroke	1. No beneficial effects were observed.<br>2. The trial was aborted.
Naeser et al. [22] (2020)	Human	–	LED;<br>633 and 870 nm<br>500 mW<br>Spot size: 22.48 cm^2<br>22.2 mW/cm^2 on the scalp	3 times per week for 6 weeks	1. tPBM (6 weeks) improved the naming ablility of chronic stroke patients when it was applied only to the left side of the head/scalp - e.g., the same side of the head, as where the stroke had occurred.<br>2. No improvement occurred when tPBM was applied to both sides of the head.
Estrada-Rojas et al. [23] (2023)	Human	–	Device 1:<br>LED; 630, 660 and 850 nm<br>Spot size: 4.9 cm^2<br>200 mW/cm^2 on the scalp<br>12 J/cm^2 on the scalp<br>Device 2:<br>LED; 810 nm; 20 min<br>24 mW/cm^2 on the scalp<br>28.8 J/cm^2 on the scalp	Usage of device 1 and device 2 together for 5 months<br>Device 1 : applied for 60 s over each of the eight, left hemisphere language network target areas<br>Device 2: applied to both hemispheres for 10 min	1. During the initial, 5-month treatment series with traditional speech-language therapy only, there was little to no improvement in dysarthria and expressive language.<br>2. tPBM plus speech-language therapy effectively improved the speech-language skills in the second, 5-month treatment series.

Table S2. Reports of tPBM used for HI in pre-clinical studies.

Authors/ year	Animal/ species	Pathological model	Parameters of the light used	Protocol	Effects
Tucker et al. [72] (2018)	Unsexed Sprague-Dawley rat pup (P10)	Unilateral permanent occlusion of the right common carotid artery following hypoxic treatment	Laser; 808 nm; 2 min<br>Spot size: 1 cm^2<br>25 mW/cm^2 on the cortex<br>3 J/cm^2 on the cortex	Once a day for 7 days	1. Decreased hemispheric brain shrinkage and neuronal cell death.<br>2. Mitigated mitochondrial fragmentation, attenuated mitochondrial membrane collapse, and enhanced ATP synthesis.<br>3. Decreased 4-Hydroxynonenal and malondialdehyde levels.<br>4. Suppressed the activation of mitochondria-dependent neuronal apoptosis.
Yang et al. [73] (2019)	Unsexed Sprague-Dawley rat pup (P10)	Unilateral permanent occlusion of the right common carotid artery following hypoxic treatment	Laser; 808 nm<br>Spot size: 1 cm^2<br>100 mW/cm^2 on the scalp<br>12 J/cm^2 on the scalp	Single treatment from 6 hours before HI	1. Alleviated memory-related behavioral deficit.<br>2. Decreased brain shrinkage volume, protected neuron and synaptic injury.<br>3. Improved mitochondrial dynamics in hippocampal CA1 neurons.<br>4. Increased the expression level of CCO in mitochondria and cytosol.<br>5. Decreased the activity of Caspase-9 and Caspase-3, and inhibited the neuronal apoptosis.
Yang et al. [71] (2021)	Unsexed Sprague-Dawley rat pup (P10)	Unilateral permanent occlusion of the right common carotid artery following hypoxic treatment	Laser; 808 nm; 2 min<br>8 mW/cm^2 on the scalp	3 times per week from gestation day 1 to gestation day 21	1. Improved survival rate and decreased infarct size.<br>2. Alleviated cortex and hippocampus related behavioral deficits.<br>3. Attenuated synaptic injury, dendritic injury, white matter injury, neuronal degeneration and neuronal apoptosis.<br>4. Preserved CCO activity and ATP production; Protected mitochondrial dynamics and mitochondrial membrane potential.<br>5. Promoted the shift of glial cells phenotype from pro-inflammatory to anti-inflammatory, and suppressed pro-inflammatory cytokines production.

Table S3. A report of tPBM used for intracerebral hemorrhage in a pre-clinical study.

Authors/ year	Animal/ species	Pathological model	Parameters of the light used	Protocol	Effects
Li et al. [74] (2023)	Male BALB/C mouse and male postnatal newborn Wistar rats	Injections of 10 μl autologous blood into the right lateral ventricle induced	Laser; 1267 nm; 51 min<br>Spot diameter: 5 mm<br>60 J/cm^2 on the scalp<br>9 J/cm^2 on the cortex	Immediately treatment: once immediately after IVH long-term treatment: once a day for 7 days	1. Immediately treatment increased the average diameter of basal MLVs and promoted the RBCs evacuation from ventricle to dcLNs.<br>2. Long-term treatment decreased intracranial pressure and mortality, and improved depression-like behavior.<br>3. tPBM-mediated stimulation of nitric oxide production improves the cleansing function of the basal MLVs.

Table S4. Reports of tPBM used for AD in pre-clinical and clinical studies.

Authors/ year	Animal/ species	Pathological model	Parameters of the light used	Protocol	Effects
Grillo et al. [83] (2013)	Female mouse (2 months old)	TASTPM transgenic model	LED; 1072 nm; 6 min 5 mW/cm^2 on the scalp 600 Hz	Once a day for 5 months	1. Increased HSP60, HSP70 and HSP105 and phosphorylated-HSP27 levels. 2. Decreased aB-crystallin, APP, tau-P, A$\beta_{1–40}$ and A$\beta_{1–42}$ levels. 3. Decreased A$\beta_{1–40/42}$ and A$\beta_{1–42}$ plaque deposition in the cortex.
Yue et al. [81] (2019)	Male mouse (4-6 months old)	APP/PS1 transgenic model	LED; 630 nm; 40 min 0.55 mW/cm^2 on the scalp	5 days per week for 2 consecutive months	1. Not only destroyed Aβ assembly but also activated formaldehyde (FA) dehydrogenase to degrade FA and attenuated FA-facilitated Aβ aggregation. 2. Smashed Aβ deposition in the extracellular space, recovered the flow of interstitial fluid, and rescued cognitive function in AD mouse models.
Zinchenko et al. [79] (2019)	Male BALB/C mouse	Injection of A$\beta_{(1-42)}$ peptide in the CA1 field	Laser; 1267 nm; 51 min Spot diameter: 5 mm 18 or 25 or 32 or 39 J/cm^2 on the cortex	Once a day for 9 days from 3 days after Aβ injection	1. tPBM with dose of 32 J/cm^2 did not induce obvious change of scalp temperature and tissue morphology. 2. Decreased cerebral Aβ burden and promoted Aβ accumulation in dcLNs. 3. Improved NSS, motor function and memory function.
Zhang et al. [80] (2020)	Male C57BL/6 mouse (6 months old)	APP/PS1 transgenic model	Laser; 635 nm; 10 min Spot size: 0.785 cm^2 2 J/cm^2 on the hippocampus and 6 J/cm^2 on the cortex	Once a day for 30 days	1. Rescued spatial learning and memory deficits. 2. Deduced Aβ production and plaque formation by shifting amyloid precursor protein (APP) processing toward the nonamyloidogenic pathway, which is CCO-dependent.
Shen et al. [82] (2021)	Male C57BL/6 mouse (6 months old)	APP/PS1 transgenic model	Laser; 635 nm; 10 min Spot size: 0.785 cm^2 2 J/cm^2 on the hippocampus and 6 J/cm^2 on the cortex	Once a day for 30 days	1. Mitigated neuronal damage, Aβ burden and neuroinflammation. 2. Rescued spatial learning and memory deficits. 3. Attenuated Aβ-induced synaptic dysfunction and neuronal death.
Semya-chkina-Glushk-ovskaya et al. [78] (2021)	Male BALB/C mouse	Injection of A$\beta_{(1-42)}$ peptide in the CA1 field	Laser; 1267 nm; 51 min Spot diameter: 5 mm 9 J/cm^2 on the scalp 3 J/cm^2 on the cortex	Once a day for 9 days from 3 days after Aβ injection	1. tPBM, especially at night, decreased cerebral Aβ burden, improved memory and learning functions, and facilitated Aβ transport from brain to dcLNs.
Tao et al. [75] (2021)	Female C57BL/6J mouse (6 or 12 months old)	APP/PS1 transgenic model	LED; 1070 nm; 6 min 25 mW/cm^2 on the scalp 10 Hz or 40 Hz (50%) 4.5 J/cm^2 on the scalp	Once a day for 60 days	1. tPBM at 10 Hz mitigated cognitive and memory impairments in 12 months old AD mouse models. 2. tPBM at 10 Hz reduced cerebral Aβ burden by activating microglia. 3. tPBM at 10 Hz decreased the number of cortical M1-like microglia that around blood vessels in 6 months old AD mouse models. 4. tPBM at 10 Hz mitigated the decrease of vascular density and length in 6 months AD mouse models by regulating VGEF level.
Lu et al. [76] (2017)	Male Sprague-Dawley rat	Injection of A$\beta_{(1-42)}$ peptide in the CA1 field	Laser; 808 nm; 2 min Spot size: 1 cm^2 25 mW/cm^2 on the cortex and 8.33 mW/cm^2 on the hippocampus	Once a day for 5 days	1. Mitigated neuronal degeneration in hippocampus CA1 region. 2. Improved mitochondrial function and dynamics. 3. Promoted CCO activity and adenosine triphosphate synthesis. 4. Suppressed glucose-6-phosphate dehydrogenase and nicotinamide adenine dinucleotide phosphate oxidase activity 5. Enhanced total antioxidant capacity and reduced oxidative damage. 6. Suppressed reactive gliosis, inflammation, and tau hyperphosphorylation.

Yang et al. [77] (2022)	Male Fischer 344 rat (2 months old)	TgF344-AD transgenic model	LED; 808 nm;<br>2 min<br>Spot size: 1.5 cm^2<br>350 mW/cm^2 on the scalp and 25 mW/cm^2 on the cortex<br>42 J/cm^2 on the scalp and 3 J/cm^2 on the cortex	3 times per week from 2 months to 18 months old	1. Rescued spatial learning and memory deficits.<br>2. Ameliorated neuronal injury, neuronal apoptosis and degeneration, and alleviated the damage to spine synapses and dendritic spines.<br>3. Attenuated amyloid load and abnormal tau hyperphosphorylation.<br>4. Recruited microglia surrounding amyloid plaques by astrocytic IL-3 and microglial IL-3Rα.<br>5. Regulated the phenotype of glial cells and suppresses neuroinflammation.<br>6. Preserved mitochondrial dynamics, and alleviated oxidative stress.<br>7. Neuronal hemoglobin mediates the neuroprotective effect of tPBM.
Berman et al. [28] (2017)	Human	–	LED; 1070 nm;<br>6 min<br>10 Hz (50 %)	Once a day for 28 consecutive days	1. Improved executive function in clock drawing, immediate recall, visual attention and task switching tests.<br>2. Not significantly improved EEG amplitude and connectivity measures.
Saltmarche et al. [25] (2017)	Human (The participants had scores from 10 to 24/30 on the MMSE at Entry.)	–	Intranasal device:<br>LED; 810 nm;<br>25 min<br>10 Hz (50 %)<br>14.2 mW/cm^2<br>Transcranial-intranasal device:<br>LED; 810 nm;<br>20 min<br>10 Hz (50 %)<br>41 mW/cm^2 (transcranial);<br>23 mW/cm^2 (intranasal)	Treated at home: once daily with the intranasal applicator only for the entire 12 weeks.<br>Received in-office: once a week, transcranial plus intranasal treatment.	1. The Mini-Mental State Exam baseline and Alzheimer's Disease Assessment Scale scores were improved after 12 weeks PBM, but precipitous declines were observed during the follow-up no-treatment, 4-week period.
Chao et al. [26] (2019)	Human	–	Transcranial-intranasal device:<br>LED; 810 nm;<br>20 min<br>40 Hz (50 %)<br>60 J/cm^2 (posterior headband);<br>45 J/cm^2 (anterior headband);<br>15 J/cm^2 (intranasal LED)	3 times per week for 12 weeks	1. Improved Alzheimer's Disease Assessment Scale-cognitive subscale and Neuropsychiatric Inventory scores after treatment.<br>2. Increased cerebral perfusion.<br>3. Increased connectivity between the posterior cingulate cortex and lateral parietal nodes within the default-mode network.
Salehpour et al. [27] (2019)	Human	–	Transcranial device:<br>LED; 635 and 810 nm; 25 min<br>Intranasal device:<br>LED; 810 nm;<br>25 min<br>10 Hz (50 %)<br>Body pad device:<br>635 and 810 nm;<br>LED; 25 min	Twice per day for 4 weeks<br>Week 1: transcranial and body pad devices PBM<br>Week 2-4: transcranial, body pad and intranasal PBM	1. Improved Montreal Cognitive Assessment score from 18 to 24 and Working Memory Questionnaire score from 53 to 10.<br>2. Mitigated olfactory dysfunction as measured by Alberta Smell test and peanut butter odor detection test.<br>3. No adverse effects were reported.

Table S5. Reports of tPBM used for PD in pre-clinical and clinical studies.

Authors/ year	Animal/ species	Pathological model	Parameters of the light used	Protocol	Effects
Shaw et al. [84] (2010)	Male BALB/C mouse (8 weeks old)	Injecting of 25 mg/kg MPTP two or four times within 30 hours induced	LED; 670 nm; 90 s Spot size: 10 cm^2 40 mW/cm^2 on the scalp	Immediately after each injection of MPTP	1. Rescued the loss of TH^+ cell in the SNc, but had no effect in ZI-Hyp.
Peoples et al. [85] (2012)	Male BALB/C mouse (8 weeks old)	Injection of 20 mg/kg MPTP two times per week for 5 weeks induced	LED; 670 nm; 90 s 5 J/cm^2 on the scalp	Immediately after each injection of MPTP (scheme 1) or in total 10 times during the survival period (scheme 2)	1. tPBM with both schemes mitigated the loss of dopaminergic cell in the SNc, but had no effect in PaG and ZI-Hyp.
Peoples et al. [86] (2012)	Male BALB/C mouse (8 weeks old)	Injecting of 25 mg/kg MPTP four times within 30 hours (acute model) or 20 mg/kg MPTP two times per week for 5 weeks (chronic model) induced	LED; 670 nm; 90 s 0.5 J/cm^2 on the cortex	Received 15 min after each MPTP injection (scheme 1) or from fourth day after the last injection (scheme 2)	1. tPBM with both schemes saved the damage of dopaminergic amacrine cell of the retina in acute and chronic PD mouse models.
Shaw et al. [87] (2012)	Male BALB/C mouse (8 weeks old)	Injection of MPTP induced acute and chronic models	LED; 670 nm; 90 s 0.5 J/cm^2 on the cortex	Received within 15 min after each MPTP injection	1. Suppressed the increase of Fos^+ cell in subthalamic nucleus and zona incerta in acute and chronic PD models, which was concurrent with the neuroprotection of TH^+ cell in the SNc.
Moro et al. [89] (2013)	Male BALB/C mouse and Male C57BL/6 mouse (8-10 weeks old)	Injecting of 25 mg/kg MPTP two times within 24 hours induced	LED; 670 nm; 90 s 0.5 J/cm^2 on the cortex	Immediately and from sixth hour after each injection of MPTP	1. Rescued the loss of TH^+ cell in the SNc in the BALB/C strain mice, but had no protection effect in the C57BL/6 strain mice.
Purushothuman et al. [94] (2013)	Male C57BL/6 mouse (5 months old)	K3 transgenic mouse model	LED; 670 nm; 90 s 4 J/cm^2 on the scalp	5 times per weeks for 4 weeks	1. Reduced oxidative stress makers (e.g. 4-HNE) and hyperphosphorylated tau levels in the SNc.<br>2. Mitigated TH^+ cell loss in the SNc.
Johnstone et al. [96] (2014)	Male BALB/C mouse (8 weeks old)	Injection of 25 mg/kg MPTP per day for 2 or 3 or 4 days induced	LED; 670 nm; 90 s 50 mW/cm^2 on the scalp 4 J/cm^2 on the scalp Delivery mode: transcranial or dorsum	Immediately (scheme 1) or from sixth day (scheme 2) after each MPTP injection	1. Dorsum tPBM with both schemes rescued TH^+ cell loss in the SNc induced by total dose of 50 mg/kg MPTP. However, this effect was not as robust as transcranial irradiation.<br>2. There was no protective effect of tPBM at a high MPTP dose (100 mg/kg in total).
Reinhart et al. [88] (2015)	Male BALB/C mouse (8-10 weeks old)	Injecting of 25 mg/kg MPTP two times within 24 hours induced	LED; 810 nm; 90 s	4 times treatment immediately (scheme 1) or from sixth hour (scheme 2) after each injecting of MPTP	1. Rescued the behavioral activity from the first treatment.<br>2. Rescued the loss of TH^+ cell in the SNc.
San Miguel et al. [90] (2019)	Male C57BL/6 mouse (12 weeks old)	Injecting of 20 mg/kg MPTP for 4 times, with a period of 2 h interval induced	LED; 670 nm; 3 min 50 mW/cm^2	Once a day for 7 days, commencing 24 h following MPTP injections	1. Mitigated MPTP-induced cerebrovascular leakage in the SNc and caudate-putamen complex at 7 days post-injection.
Oueslati et al. [93] (2015)	Female Sprague-Dawly rat	Adeno associated virus based genetic model	Laser; 808 nm; 100 s Spot size: 2 cm^2 2.5 or 5 mW/cm^2	Once a day for 28 days	1. tPBM with dose of 0.5 J/cm^2 suppressed motor deficit, rescued the loss of TH^+ cell in the SNc, and preserved dopaminergic fiber in the ipsilateral striatum.<br>2. Above beneficial effects were still existing 42 days after treatment.

Salgado et al. [92] (2016)	Wistar rat	Bilateral microinjections 6-OHDA into the substantia induced	LED; 627 nm; 57 s 4 J/cm^2 on the scalp or Laser: 630 nm 88 s 4 J/cm^2 on the scalp	Once a day for 7 days from 30 days after injection of 6-OHDA	1. Both laser and LED irradiation improved depression-like behavior, with LED being the better. 2. Laser treatment increased serum IL-2 level and decreased serum TNF-α and INF-γ levels. 3. LED treatment decreased serum TNF-α level, but not as robust as laser; LED treatment increased serum INF-γ level.
O'Brien et al. [91] (2019)	Male Sprague Dawley rat	Injection of LPS induced	LED; 670 nm; 88 s Spot size: 10 cm^2 40.8 mW/cm^2 on the scalp 3.59 J/cm^2 on the scalp	Twice a day for 6 days after injection	1. Prevented dopaminergic cell loss in SNc induced by 10 µg LPS instead of 20 µg LPS. 2. Behavior improvement was not related to the loss of dopaminergic cell in the SNc.
Darlot et al. [95] (2016)	Male macaque monkey (4 to 5 years old)	Injecting of MPTP induced	Laser; 670 nm Implantable fiber 10 mW Total dose of 25 J (Dose 1) or 35 J (Dose 2)	Received within 24 hours after each MPTP injection	1. tPBM, especially total dose of 25 J, rescued clinical evaluation scores and improved behavioral activity. 2. tPBM, especially total dose of 25 J, mitigated the loss of TH$^+$ cell and striatum TH$^+$ terminal in the bilateral SNc.
Hamilton et al. [31] (2019)	Human	–	LED; A helmet contains several LEDs (660, 810 and 850 nm)	1 or 2 times per day	1. 55% of the initial signs and symptoms of the six patients showed overall improvement, whereas 43% stayed the same and only 2% worsened after tPBM treatment.
Liebert et al. [29] (2021)	Human (aged from 60 to 80)	–	LED; transcranial, intranasal, neck and abdominal PBM device containing several LEDs	3 times per week for week 1 to 4; 2 times per week for week 5 to 8; and once per week for week 9 to 12	1. Measures of mobility, cognition, dynamic balance and fine motor skills were improved with tPBM treatment for 12 weeks and up to one year.
Hong et al. [32] (2021)	Human (aged from 58 to 80)	–	LED; 940 nm; 30 min 6 mW/cm^2	5 times per week for 2 weeks	1. Improved Unified Parkinson Disease Rating Scale scores, and the improvement persisted until the end of treatment. 2. The beneficial effects still partially existed one week after the treatment stopped.
Liebert et al. [30] (2022)	Human	–	Laser; 904 nm Abdomen-neck PBM (brain-gut axis)	3 times per week for 12 weeks	1. Improved a number of clinical signs of PD, including mobility, cognition, dynamic balance, spiral test, and sense of smell, with some being maintained for 45 weeks. 2. Improvements varied from person to person.
McGee et al. [33] (2023)	Human	–	LED; 635 and 810 nm; 24 min	6 times per week for 12 weeks	1. Improved facial and lower-limb scores in MDS-UPDRS-III test.
Liebert et al. [34] (2023)	Human	–	Device 1: Laser; 904 nm 50 Hz; 11 min 60 mW Device 2: LED; 670 and 810 nm; 12 min	Usage of device 1 and device 2 together for 3 years	1. Median values for mobility and cognition continued to improve to 2 years and slightly declined to 3-years. 2. Participants who discontinued treatment after 1 year showed a decline in outcome measures.

Table S6. Reports of tPBM used for MS in pre-clinical and clinical studies.

Authors/ year	Animal/ species	Pathological model	Parameters of the light used	Protocol	Effects
Duarte et al. [98] (2018)	Male C57BL/6 mouse (7 weeks old)	Injection of cuprizone induced	Laser; 808 nm; 20 s<br>Spot size: $0.028\ cm^2$<br>$1.78\ W/cm^2$<br>$36\ J/cm^2$	Once a day for 3 days in the third and fourth weeks of cuprizone administration	1. Improved motor coordination ability.<br>2. Inhibited the increase of serum lactate dehydrogenase.<br>3. Attenuated demyelination.<br>4. Attenuated the activation of glia cell.
Silva et al. [97] (2020)	Human	–	Laser; 808 nm; 360 s<br>Spot size: $0.127\ cm^2$<br>$0.8\ W/cm^2$<br>$287\ J/cm^2$	Twice a week for 12 weeks	1. Increased the level of serum proinflammatory cytokine IL-10.
Silva et al. [99] (2022)	Human	–	Laser; 808 nm; 360 s<br>Spot size: $0.127\ cm^2$<br>$0.8\ W/cm^2$<br>$287\ J/cm^2$	Twice a week for 12 weeks	1. Not significantly improved fatigue status measured by modified fatigue impact scale.

Table S7. Reports of tPBM used for TBI in pre-clinical and clinical studies.

Authors/ year	Animal/ species	Pathological model	Parameters of the light used	Protocol	Effects
Oron et al. [114] (2007)	Male sabra mouse	Closed head impact (CHI) (NSS 4-6)	Laser; 808 nm; 2 min Spot diameter: 1.2 cm 10 (Dose 1) or 20 (Dose 2) mW/cm^2 on the cortex	Single treatment from 4 hours after TBI	1. tPBM with both doses improved NSS without significant difference observed. 2. Decreased the size of lesion region.
Ando et al. [101] (2011)	Male BALB/C mouse	Controlled cortical impact (CCI)	Laser; 810 nm; 12 min 50 mW/cm^2 on the scalp 36 J/cm^2 on the scalp CW or PW 1(10 Hz) or PW 2(100 Hz)	Single treatment from 4 hour after TBI	1. tPBM (PW 2) improved NSS. 2. tPBM (PW 2) improved motor function and mitigated depression-like behavior. 3. tPBM (PW 2) not significantly recovered ATP level in trauma region. 4. tPBM (PW 1) mitigated the size of brain lesion region.
Oron et al. [115] (2012)	Male sabra mouse (2 months old)	CHI	Laser; 808 nm; 2 min Spot diameter: 1.2 cm 10 mW/cm^2 on the cortex 1.2 J/cm^2 on the cortex CW or PW (100 or 600 Hz)	Single treatment	1. Both PW and CW modes of treatment decreased the size of lesion region and improved NSS. 2. 100 Hz-PW light had a better NSS improvement than both 600 Hz-PW and CW light treatment.
Khuman et al. [112] (2012)	Male C57BL/6 mouse (3 months old)	CCI with craniotomy	Laser; 800 nm Spot size: 1.32 cm^2 Craniotomy: 30 J/cm^2 (dose 1) or 60 J/cm^2 (dose 2) or 105 J/cm^2 (dose 3) or 120 J/cm^2 (dose 4) or 210 J/cm^2 (dose 5) on the cortex Transcranial: 60 J/cm^2 (dose 6) on the cortex	Once a day for 1 (scheme 1) or 7 days (scheme 2) from 60-80 min after TBI	1. Single craniotomy and transcranial laser irradiation with dose of 60 J/cm^2 improved motor and memory functions. 2. Single craniotomy and transcranial laser irradiation with dose of 60 J/cm^2 inhibited the activation of microglia, but had no beneficial effects on lesion size, edema degree and brain nitrotyrosine level.
Wu et al. [102] (2012)	Male BALB/C mouse	CHI	Laser; 4 min 660 or 730 or 810 or 980 nm 150 mW/cm^2 on the scalp 36 J/cm^2 on the scalp	Single treatment from 4 hours after TBI	1. tPBM at 660 and 810 nm improved NSS during follow up period. 2. tPBM with 660 and 810 nm decreased the size of brain lesion region.
Xuan et al. [103] (2013)	Male BALB/C mouse (6-8 weeks old)	Moderate to severe cortical impact (NSS 7-8) with craniotomy	Laser; 810 nm; 12 min Spot diameter: 1 cm 25 mW/cm^2 on the scalp 18 J/cm^2 on the scalp	Single treatment (scheme 1) or once a day for 3 days (scheme 2) or once a day for 14 days (scheme 3) from 4 hours after TBI	1. tPBM for 3 days improved NSS, but got worse for 14 days. 2. tPBM for 3 days improved motor function in wire-grip and motion tests. 3. tPBM for 3 days decreased the size of lesion region. 4. tPBM for 3 days mitigated neuronal degeneration and promoted neurogenesis from 4 weeks after TBI.
Xuan et al. [104] (2014)	Male BALB/C mouse (8 weeks old)	Moderate to severe cortical impact (NSS 7-8) with craniotomy	Laser; 810 nm; 12 min Spot diameter: 1 cm 25 mW/cm^2 on the scalp 18 J/cm^2 on the scalp	Single treatment (scheme 1) or once a day for 3 days from 4 hours after TBI (scheme 2)	1. tPBM (scheme 2) improved motor and memory functions. 2. tPBM (scheme 2) inhibited the level of Caspase-3 in lesion region from 4 days after TBI. 3. tPBM (scheme 2) increased the number of BrdU/NeuN$^+$ cells in the hippocampus from 28 days after TBI. 4. tPBM (scheme 2) increased the expression level of neuron tubulin (e.g. DCX and TUJ-1) in the hippocampus.
Zhang et al. [108] (2014)	Wild-type mouse and IEX-1	Mild cortical impact (NSS	Laser; 810 nm; 4 min 10 Hz (50%)	Single treatment from 4 hours after TBI	1. Downregulated the level of IL-6, but upregulated the level of TNF-α. 2. Rescued NSS and the size of brain damage.

	knockout mouse (8 weeks old)	~5) with craniotomy	150 mW/cm^2 on the scalp<br>36 J/cm^2 on the scalp		3. Inhibited apoptosis and leukocyte infiltration in the cortex and hippocampus.<br>4. Increased ATP level in the impact region, which is correlated to NSS.
Xuan et al. [105] (2015)	Male BALB/C mouse (6-8 weeks old)	Moderate to severe cortical impact (NSS 7-8) with craniotomy	Laser; 810 nm; 12 min<br>Spot diameter: 1 cm<br>50 mW/cm^2 on the scalp<br>36 J/cm^2 on the scalp	Single treatment (scheme 1) or once a day for 3 days (scheme 2) from 4 hours after TBI	1. tPBM (scheme 2) increased the BDNF level in the DG and SVZ regions 7 days after TBI.<br>2. tPBM (scheme 2) increased the expression of synapsin-1 in the lesion cortex and SVZ.
Dong et al. [109] (2015)	C57BL/6 mouse (8 weeks old)	CHI (NSS 5-6)	Laser; 810 nm; 4 min<br>10 Hz (50%)<br>150 mW/cm^2 on the scalp<br>36 J/cm^2 on the scalp	Single treatment from 4 hours after TBI	1. tPBM combined with lactic acid or pyruvate treatment further increased ATP level, but did not affect ROS level.<br>2. tPBM combined with lactic acid or pyruvate treatment further decreased the size of lesion area, improved the memory function, and mitigated neuronal injury and inflammation.
Xuan et al. [106] (2016)	Male BALB/C mouse (6-8 weeks old)	Moderate to severe cortical impact (NSS 7-8) with craniotomy	Laser; 810 nm; 12 min<br>Spot diameter: 1 cm<br>25 mW/cm^2 on the scalp<br>18 J/cm^2 on the scalp	Once a day for 3 days (scheme 1) or 14 days (scheme 2) from 4 hours after TBI	1. tPBM (scheme 1) improved neurological scores, memory function and the size of lesion, but got worse with scheme 2.<br>2. tPBM (scheme 2) temporarily inhibited the brain repair process by increasing the number of activated astrocytes.
Shemesh et al. [107] (2022)	Female mouse (12 weeks old)	CHI	Laser; 810 nm; 15 min<br>Spot size: 4 cm^2<br>50 mW/cm^2 on the scalp<br>45 J/cm^2 on the scalp	Single treatment from 10 min after TBI	1. Inhibited the development of complications in the injured mouse by increasing blood flow, blood saturation and overall oxygen consumption levels over the injured area.
Moreira et al. [113] (2009)	Male Wista rat	Cortex cryogenic brain trauma	Laser;<br>660 or 780 nm<br>PW mode<br>3 J/cm^2 (dose 1) or 5 J/cm^2 (dose 2)	Double treatment: one immediately after brain injury and the other 3 hours later	1. 660 nm-tPBM with dose 2 decreased IL-1β level in brain tissue, but 780 nm-tPBM with dose 1 increased its level.<br>2. tPBM (660 nm with both dose, and 780 nm with dose 2) increased serum TNF-α and IL-6 levels.
Quirk et al. [111] (2012)	Sprague-Dawley rat	CCI with craniotomy	LED; 670 nm; 5 min<br>50 mW/cm^2 on the scalp<br>1.5 J/cm^2 on the scalp	Twice a day for 3 days (biochemical analysis) or 10 days (behavior test)	1. Improved motor function.<br>2. Decreased Bax pro-apoptotic marker, increased Bcl-2 anti-apoptotic marker, and reduced glutathione level.
Esenaliev et al. [100] (2018)	Male Sprague-Dawley rat	Blast brain injury induced	Laser; 808 nm; 5 min<br>300 J/cm^2 on scalp<br>PW mode: 20 Hz	Single treatment from 1 hour after TBI	1. Improved motor function.<br>2. Downregulated apoptosis genes (e.g. Bax, Caspase-3) and increased BDNF expression in the cortex and hippocampus.<br>3. Inhibited the activation of microglia and promoted the proliferation of neuronal progenitor cells from 10 days after TBI in the cortex.
Mocciaro et al. [110] (2020)	Male Sprague-Dawley rat	Fluid percussion injury with craniotomy	Laser; 808 nm; 5 min<br>300 J/cm^2 on the scalp<br>PW mode: 20 Hz	Single treatment from 1 hour after TBI	1. Improved the motor and cognitive functions.<br>2. Decreased impaired maturation and aberrant migration of neural progenitors in the DG.<br>3. Prevented upregulation of specific microRNAs in neural stem cells.
Yang et al. [116] (2020)	Male Sprague-Dawley rat	Repeated CHI (three times, with an interval of 5 days)	Laser; 808 nm; 2 min<br>25 mW/cm^2 on the cortex<br>350 mW/cm^2 on the scalp	Single treatment for 15 days from 2 hours after TBI	1. Improved NSS, motor function, depression-like behavior, memory and social interaction.<br>2. Mitigated the downregulation of synaptic proteins (e.g. synaptophysin).<br>3. Inhibited neuronal injury in the cortex and hippocampus.<br>4. Inhibited gliosis and increased the IL-1β and IL-10 levels.<br>5. Increased the number of NeuN$^+$ cells, decreased the number of TUNEL$^+$ cells, and decreased the activity of Caspase-9 and

					Caspase-3.
Naeser et al. [38] (2011)	Human	Chronic TBI	LED arrays including several red diodes (633 nm) and NIR diodes (870 nm)	Once a week for 8 weeks (patient 1)<br>Once a day for 9 months (patient 2)	1. tPBM for 8 weeks improved attention time from 20 min to 3 hours.<br>2. tPBM for 9 months improved executive and memory functions, and reduced the post-traumatic stress disorders.
Nawashiro et al. [40] (2012)	Human	Severe head trauma	LED; 850 nm;<br>30 min<br>Spot size: 21.85 cm^2<br>11.4 mW/cm^2 on the scalp<br>20.5 J/cm^2 on the scalp	Twice a day for 73 days from 228 days after TBI	1. tPBM for 5 days improved motor function.<br>2. tPBM for 73 days increased 20% local CBF in left prefrontal lobe.
Naeser et al. [36] (2014)	Human	Mild TBI	A device contains 9 red diodes (633 nm) and 52 NIR diodes (870 nm); 10 min	3 times per week for 6 weeks	1. Improved executive function and cognitive performance.
Naeser et al. [37] (2016)	Human	Chronic TBI	tPBM:<br>LED<br>Intranasal PBM:<br>Red LED<br>(633 nm<br>8 mW/cm^2) and<br>NIR LED<br>(810 nm<br>14.2 mW/cm^2)	3 times per week for 6 weeks	1. Both tPBM and intranasal PBM treatments improved sleep (on average one additional hour per night) in chronic TBI cases.
Chao et al. [35] (2020)	Human	Moderate TBI	LED; 810 nm;<br>10 min<br>Power density of posterior, anterior and intranasal LEDs are 100, 75, and 25 mW/cm^2 respectively.<br>PW mode:<br>10 Hz (device 1)<br>40 Hz (device 2)	Every other day for 8 weeks, alternating device 1 and device 2 depending on the patient's response	1. Improved language learning, memory and executive functions; mitigated headache symptom.<br>2. Enhanced brain functional connectivity.<br>3. Increased the perfusion in gray matter, frontal lobe, temporal lobe, occipital lobe and hippocampus.
Figueiro Longo et al. [39] (2020)	Human	Moderate TBI	LED; 810 nm;<br>20 min<br>36 mW/cm^2 on the scalp<br>43 J/cm^2 on the scalp	Triple treatments from 72 hours after TBI, each more than 12 hours apart	1. Promoted neuroreactivity in white matter tracts.<br>2. No adverse reactions were observed.

Table S8. A report of tPBM used for Possible CTE in a clinical study.

Authors/ year	Patients information	Device	Protocol	Effects
Naeser et al. [117] (2023)	American-style ex-football players<br>Case 1:<br>65 years old with emotional outbursts, depression, cognitive problems, poor memory and poor sleep<br>Case 2:<br>55 years old with emotional outbursts, cognitive problems and poor memory<br>Case 3:<br>57 years old with emotional outbursts, depression, cognitive problems, poor memory, poor sleep, chronic pain and tinnitus<br>Case 4:<br>74 years old with emotional outbursts, cognitive problems and chronic pain	Device A:<br>In-Office<br>MedX Health LED device<br>(633 and 870 nm; 40 min)<br>Device B:<br>At-Home<br>Neuro Gamma device<br>(810 nm; 20 min) and Vielight intranasal LED device (633 nm; 25 min)<br>Device C:<br>In-Office<br>THOR Photomedicine LED lined helmet<br>(660 and 850 nm; 23 min)	Treated with device A or C 3 times per week for 6 weeks, followed by device B treatment at home, 6 times per week	1. Improved executive function, attention, PTSD, pain and sleep. This was significantly correlated to the increase of SN functional connectivity.<br>2. Improved verbal learning/memory and depression. This was correlated to the increase of CEN functional connectivity.<br>3. Increased n-acetyl-aspartate (oxygen consumption, mitochondria) in anterior cingulate cortex. This was parallel to less pain and PTSD.

Table S9. Reports of tPBM used for depression in pre-clinical and clinical studies.

Authors/ year	Animal/ species	Pathological model	Parameters of the light used	Protocol	Effects
Xu et al. [121] (2017)	Male ICR mouse	Movement restriction induced and Abelson helper integration site-1 knockout model	Laser; 808 nm; 30 min Spot diameter: 1 cm 23 mW/cm^2 on the scalp	Once a day for 28 days	1. Improved depression-like behavior. 2. Rescued the decline of ATP level in prefrontal cortex region. 3. Increased the activity and level of mitochondrial complex IV in the prefrontal cortex.
Salehpour et al. [118] (2018)	Male BALB/C mouse	Sleep deprivation induced	Laser; 810 nm; 5 s PW mode: 10 Hz (88%) Spot size: 0.03 cm^2 8 J/cm^2 on the cortex	Once a day for 3 days	1. Prevented cognitive impairment. 2. Enhanced the antioxidant status and increased mitochondrial activity in the hippocampus.
Salehpour et al. [119] (2019)	Male BALB/C mouse (8-10 weeks old)	Subchronic restraint stress induced	Laser; 810 nm; 5 s 10 Hz (88%) Spot size: 0.03 cm^2 33.3 J/cm^2 on the scalp	Once a day for 5 days	1. Improved depression-like behavior. 2. Decreased lipid peroxidation. 3. Enhanced total antioxidant capacity and glutathione levels, glutathione peroxidase and superoxide dismutase activity. 4. Decreased nuclear factor kB, p38, and JNK levels in the hippocampus and cortex. 5. Downregulated intrinsic apoptosis biomarkers (e.g. BAX, Bcl-2, and caspase-3). 6. Decreased serum cortisol, corticosterone, TNF-α, and IL-6 levels.
Farazi et al. [120] (2022)	Male BALB/C mouse	110 dB white noise induced	Laser; 810 nm; 5 s PW mode: 10 Hz (88%) 4.75 W/cm^2 on the scalp 8 J/cm^2 on the cortex	Once a day for 14 days	1. Improved depression-like behavior in forced swimming, elevated plus maze and open field tests. 2. Upregulated the BDNF/tyrosine receptor kinase B/CREB signaling pathway in the hippocampus. 3. Reduced serum corticosterone level.
Wu et al. [122] (2012)	Male Wistar rat	Variety of mild unpredictable stressors for a total of 8 weeks induced	Laser; 810 nm; 2 min 15 mW/cm^2 on the cortex PW mode: 100 Hz (20%)	3 times per week for 3 weeks	1. Improved depression-like behavior without significant difference compared to fluoxetine treatment.
Mohammed et al. [124] (2016)	Male Rattus norvegicus rat	Injection of reserpine induced	Laser; 804 nm; 6 min Spot size: 0.13 cm^2 0.64 or 1.60 or 3.18 mW/cm^2	Once a day for 14 days	1. tPBM (0.64 W/cm^2) decreased immobile time, and increased swimming and climbing abilities. 2. tPBM (3.18 W/cm^2) deteriorated behavior function. 3. tPBM (0.64 W/cm^2) mitigated the decline of Delta frequency band and increase of Beta-1 and Beta-2 frequency bands.
Salehpour et al. [123] (2016)	Male Wistar rat (12 weeks old)	Chronic mild stress model induced by exposing to stressors over several weeks	Laser; 630 or 810 nm Spot size: 0.07 cm^2 PW mode: 10 Hz (50%) 1.18 J/cm^2 on the cortex for both wavelengths	4 times per week for 3 weeks	1. tPBM (810 nm) improved depression-like behavior and elevated body weight. 2. tPBM decreased serum cortisol level and increased blood sugar level.
Li et al. [125] (2021)	Male Sprague-Dawley rat	PTSD model induced by underwater trauma	Laser; 808 nm Spot size: 1.5 cm^2 25 mW/cm^2 on the cortex 3 J/cm^2 on the cortex	Once a day for 7 days	1. Improved anxiety-like behavior, depression-like behavior, and cognitive dysfunction. 2. Regulated Arc and c-fos expression in the hippocampus and amygdala. 3. Boosted ATP production and regulated protein expression in the hippocampus.
Yang et al. [126] (2021)	Male Fischer 334 rat	TgF344-AD transgenic model	Laser; 808 nm; 2 min 350 mW/cm^2 on the scalp and 25	3 times per week for 8 months from age of 2 months	1. Improved anxiety and depression-like behavior. 2. Inhibited neuronal apoptosis and damage in the cortex. 3. Alleviated mitochondria fragmentation and

			mW/cm^2 on the cortex<br>42 J/cm^2 on the scalp and 3 J/cm^2 on the cortex		rescued the decline of CCO activity and ATP level.<br>4. Inhibited the activation of microglia, and decreased the expression of proinflammatory cytokines (e.g. TNF-α).<br>5. Enhanced total antioxidant capacity and reduced the expression of malondialdehyde in the cortex.
Schiffer et al. [42] (2009)	Human	–	LED; 810 nm<br>4 positions each for 4 min<br>250 mW/cm^2 on the scalp and 9.5 mW/cm^2 on the cortex	2 weeks (scheme 1) or 4 weeks (scheme 2)	1. tPBM (scheme 1) not significantly increased mean rCBF across hemispheres.<br>2. tPBM (scheme 2) improved HAM-D and HAM-A.
Disner et al. [41] (2016)	Human	–	Laser; 1064 nm; 4 min<br>Spot size: 13.6 cm^2<br>250 mW/cm^2 on the scalp<br>60 J/cm^2 on the scalp	Double treatments with 48 hours apart	1. Enhanced the therapeutic effect of ABM.
Cassano et al. [43] (2018)	Human	–	LED; 823 nm; 20-30 min<br>Spot size: 28.7 cm^2<br>33.2 mW/cm^2	Twice a week for 8 weeks	1. Improved the baseline observation carried forward, last observation carried forward based on the change in HAM-D_{17} total scores.
Kerppers et al. [44] (2020)	Human	–	LED; 945 nm; 85 s<br>9.25 J/cm^2 on the scalp	Once a day for 30 days	1. Improved HAD scale scores.<br>2. Improved the performance in memory and drawings tests, but had no beneficial effect in strength test.

Table S10. Reports of tPBM used for aging in pre-clinical and clinical studies.

Authors/ year	Animal/ species	Pathological model	Parameters of the light used	Protocol	Effects
Michalikova et al. [131] (2008)	Female CD1 mouse	Natural aging (12 months old)	Laser; 1072 nm; 6 min	Once a day for 10 days	1. Rescued the memory impairment in 3D-maze test.
Salehpour et al. [127] (2017)	Male BALB/C mouse	Injection of D-galactose induced	Laser; 660 or 810 nm PW mode: 10 Hz (88%) 4.75 W/cm^2 on the scalp 4 or 8 J/cm^2 on the cortex surface	3 times per week during model establishment period	1. Red and NIR laser at high dose (8 J/cm^2) mitigated cognitive impairment and improved active mitochondria level, mitochondrial membrane potential level, ATP level, and CCO activity. 2. Red and NIR laser at high dose (8 J/cm^2) decreased Bax/Bcl-2, Caspase-3 and ROS levels.
Massri et al. [132] (2018)	Male C57BL/6 mouse	Natural aging (12 months old)	LED; 670 nm; 20 min	Once a day for 8 months from age of 5 months	1. Reduced the number and volume of striatal astrocytes. 2. Reduced the number of microglia in striatum.
Salehpour et al. [130] (2018)	Male BALB/C mouse	Natural aging (18 months old)	Laser; 660 nm; 15 s Spot size: 0.03 cm^2 6.66 W/cm^2 on the scalp 99.9 J/cm^2 on the scalp	Once a day for 2 weeks	1. Improved spatial memory and anxiety-like behaviors. 2. Increased ATP level in the hippocampus.
Salehpour et al. [128] (2019)	Male BALB/C mouse	Injection of D-galactose induced	Laser; 660 nm; 15 s Spot size: 0.03 cm^2 6.66 W/cm^2 on the scalp 99.9 J/cm^2 on the scalp and 16 J/cm^2 on the cortex	Once a day for 2 weeks	1. Improved spatial memory and anxiety-like behaviors. 2. Increased ATP level in the hippocampus.
Hosseini et al. [129] (2022)	Male BALB/C mouse	Injection of D-galactose and aluminum chloride induced	Laser; 810 nm Spot size: 0.03 cm^2 4.75 W/cm^2 on the scalp 8 or 16 or 32 J/cm^2 on the cortex	3 times per week for 2 months	1. Mitigated social and spatial memory impairments. 2. Inhibited the down-regulation of growth-associated protein 43 and synaptophysin. 3. Decreased the expression of proinflammatory cytokines (e.g., TNF-α and IL-6).
Cardoso et al. [134] (2021)	Male Wistar rat	Natural aging (20 months old)	Laser; 810 nm; 30 s Spot size: 0.028 cm^2 3.57 W/cm^2 on the scalp and 17 mW/cm^2 on the cortex 107 J/cm^2 on the scalp	Once a day for 58 days	1. Increased cortical intracellular signaling proteins related to cell proliferation and cell survival. 2. Increased the expression of p70 ribosomal protein S6 kinase, signal transducer, activator of transcription 3 and the activation of Akt in the hippocampus.
Cardoso et al. [133] (2022)	Male Wistar rat	Natural aging (20 months old)	Laser; 810 nm; 30 s Spot size: 0.025 cm^2 4.06 W/cm^2 on the scalp	Once a day for 58 days	1. Reversed age-related decrease in regional brain CCO activity and the impairment of systems-level functional connectivity.
Cardoso et al. [135] (2022)	Male Wistar rat	Natural aging (20 months old)	Laser; 810 nm; 30 s Spot size: 0.028 cm^2 3.57 W/cm^2 on the scalp and 17 mW/cm^2 on the cortex 107 J/cm^2 on the scalp	Once a day for 28 days	1. Improved spatial learning and memory abilities. 2. Increased IL-6, TNF-α and IL-10 levels and decreased IL-5 level in the cortex. 3. Decreased IP-10 and fractalkine levels in the hippocampus.
Cardoso et al.	Male Wistar rat	Natural aging (20 months old)	Laser; 660 nm; 30 s Spot size:	Once a day for 10 days	1. Increased IL-1α level and decreased IL-5 level in the cortex. 2. Increased IL-1α level and decreased IL-5,

[136] (2022)			0.03 cm^2<br>3.33 mW/cm^2		IL-18 and fractalkine levels in the hippocampus.<br>3. Activated intracellular signaling proteins linked to vascular function and cell survival.
Salgado et al. [46] (2015)	Human	Aged over 60	LED; 627 nm<br>70 mW/cm^2 on the scalp<br>40 J/cm^2 on the cortex	Twice a week for 4 weeks	1. Increased systolic and diastolic velocity of the left middle cerebral and basilar arteries.<br>2. Decreased pulsatility index and resistance index in the cerebral arteries.
Vargas et al. [45] (2017)	Human	Aged 49-90	Laser; 1064 nm<br>Spot size: 13.6 cm^2<br>250 mW/cm^2 on the forehead<br>120 J/cm^2 on the forehead	Once a week for 5 weeks	1. Mitigated attention and memory impairments.<br>2. Increased resting-state EEG alpha, beta, and gamma power.<br>3. Promoted more efficient prefrontal BOLD fMRI response.
Chan et al. [48] (2019)	Human	Aged over 60	A device contains several red diodes (633 nm) and NIR diodes (870 nm); 7.5 min<br>Spot size: 22.48 cm^2<br>44.4 mW/cm^2 on the scalp<br>20 J/cm^2 on the scalp	Single treatment	1. Improved action selection and inhibition abilities in flanker test.<br>2. Improved mental by flexibility flanker test.
Chan et al. [49] (2021)	Human	Not mentioned	LED; 810 nm; 350 s<br>20 mW/cm^2 on the scalp<br>7 J/cm^2 on the scalp	Single treatment	1. Improved visual memory performance and reduced the hemodynamic response during the test.
Saucedo et al. [47] (2021)	Human	Aged 45-85	Laser; 1064 nm<br>Spot size: 13.6 cm^2<br>250 mW/cm^2 on forehead<br>120 J/cm^2 on forehead	Single treatment	1. Increased CCO level during irradiation, followed by a significant post simulation increase in oxygenated hemoglobin and a decrease in deoxygenated hemoglobin.
Qu et al. [50] (2022)	Human	Aged 50-77	Laser; 1064 nm; 12 min<br>0.25 W/cm^2	Single treatment (scheme 1) or once a day for 7 consecutive days (scheme 2)	1. tPBM with both schemes improved working memory of older adults, and the improvement of scheme 2 was better.<br>2. The beneficial effect of scheme 2 lasted at least 3 weeks.

Table S11. Reports of tPBM used for epilepsy in pre-clinical studies.

Authors/ year	Animal/ species	Pathological model	Parameters of the light used	Protocol	Effects
Hong et al. [141] (2022)	Male C57BL/6 mouse (7 weeks old)	Injection of pilocarpine induced	Laser; 830 nm; 12 min 50 mW/cm^2 on the scalp 22.9 J/cm^2 on the scalp	Single treatment (scheme 1) or once a day for 5 days (scheme 2)	1. tPBM (scheme 2) increased hilar interneuron population in the DG. 2. tPBM (scheme 2) increased Ki-67$^+$ cell expression level in the DG 7 and 14 days after status epilepticus. 3. tPBM (scheme 2) increased the number of NeuN$^+$/BrdU$^+$ and DCX$^+$ cells in the DG 14 days after status epilepticus.
Radwan et al. [138] (2009)	Male Rattus norvegicus rat	Injection of pilocarpine induced	Laser; 830 nm; 6 min 90 mW 32.4 J	Once a day for 7 days	1. Returned the concentrations of glutamic acid, glutamine, glycine, and taurine to initial levels in the cortex. 2. Returned the concentrations of aspartate, glycine to near-control values. 3. Increased aspartate aminotransferase (ALT), alanine aminotransferase (AST) activity and glucose content in the cortex. 4. Increased ALT and AST activity. 5. Decreased the concentrations of glucose in the hippocampus.
Tsai et al. [137] (2020)	Sprague-Dawley rat (p30-36)	Subcutaneous injection of PTZ induced	Laser; 808 nm; 100 s Spot size: 0.0825 cm^2 1.333 W/cm^2 on the scalp 133.3 J/cm^2 on the scalp	Single treatment before PTZ injection	1. Reduced mean seizure scores, incidence of status epilepticus and mortality. 2. Reduced dark neurons in the cortex, hippocampus, thalamus and hypothalamus. 3. Lessened the apoptotic ratio of parvalbumin (PV)-positive interneurons and alleviated the aberrant extent of PV-positive unstained soma of principal cells in the hippocampus.
Vogel et al. [139] (2021)	Male Wistar rat	Photothromboti c model	Laser; 780 nm; 2 min Spot size: 0.12 cm^2 0.083 W/cm^2 on the scalp 10 J/cm^2 on the scalp	3 times per week for 8 weeks	1. Reduced electrographic seizure duration and spikes number in the cortex and ventral posteromedial thalamic nucleus.
Tsai et al. [140] (2022)	Sprague-Dawley rat (p30-36)	Subcutaneous injection of PTZ induced	Laser; 808 nm; 100 s Spot size: 0.0825 cm^2 1.333 W/cm^2 on the scalp 133.3 J/cm^2 on the scalp	Single treatment before PTZ injection	1. Reduced neuron-specific enolase immunoreactivity in the hippocampus CA3. 2. Inhibited the activation of microglia and astrocytes. 3. Enhanced hippocampal CCO oxidase subunit 1.
